# Attaining expert consensus on diagnostic expectations of primary chronic pain diagnoses for patients referred to interdisciplinary pediatric chronic pain programs: A delphi study with pediatric chronic pain physicians and advanced practice nurses

**DOI:** 10.3389/fpain.2022.1001028

**Published:** 2022-09-12

**Authors:** Megan Greenough, Tracey Bucknall, Lindsay Jibb, Krystina Lewis, Christine Lamontagne, Janet Elaine Squires

**Affiliations:** ^1^ School of Nursing, Chronic Pain Services, The Children’s Hospital of Eastern Ontario Chronic Pain Services at The Children’s Hospital of Eastern Ontario, University of Ottawa, Ottawa, ON, Canada; ^2^ School of Nursing, Deakin University, Centre for Quality and Patient Safety Research, Institute for Health Transformation, Geelong, VIC, Australia; ^3^ Bloomberg Faculty of Nursing, Pediatric Nursing Research, SickKids Hospital, University of Toronto, Toronto, ON, Canada; ^4^ School of Nursing, The University of Ottawa, Ottawa, ON, Canada; ^5^ Department of Medicine, Chronic Pain Services, The Children’s Hospital of Eastern OntarioUniversity of Ottawa, Ottawa, ON, Canada; ^6^ School of Nursing, University Research Chair in Health Evidence Implementation, University of Ottawa, The Ottawa Hospital Research Institute, Ottawa, ON, Canada

**Keywords:** chronic pain, interdisciplinary chronic pain program, referral practices, pediatric, diagnostic investigations, significant clinical indicators, red flags

## Abstract

**Objective:**

Pediatric primary chronic pain disorders come with diagnostic uncertainty, which may obscure diagnostic expectations for referring providers and the decision to accept or re-direct patients into interdisciplinary pediatric chronic pain programs based on diagnostic completeness. We aimed to attain expert consensus on diagnostic expectations for patients who are referred to interdisciplinary pediatric chronic pain programs with six common primary chronic pain diagnoses.

**Method:**

We conducted a modified Delphi study with pediatric chronic pain physicians, nurse practitioners and clinical nurse specialists to determine degree of importance on significant clinical indicators and diagnostic items relevant to each of the six primary chronic pain diagnoses. Items were identified through point of care databases and complimentary literature and were rated by participants on a 5-point Likert scale. Our consensus threshold was set at 70%.

**Results:**

Amongst 22 experts across 14 interdisciplinary programs in round one and 16 experts across 12 interdisciplinary programs in round two, consensus was reached on 84% of diagnostic items, where the highest degree of agreement was with Complex Regional Pain Syndrome (CRPS), Type 1 (100%) and the lowest with chronic pelvic pain (67%).

**Conclusion:**

This study demonstrated a general agreement amongst pediatric chronic pain experts regarding diagnostic expectations of patients referred to interdisciplinary chronic pain programs with primary chronic pain diagnoses. Study findings may help to clarify referral expectations and the decision to accept or re-direct patients into such programs based on diagnostic completeness while reducing the occurrence of unnecessary diagnostic tests and subsequent delays in accessing specialized care.

## Introduction

Chronic pain in children and adolescents is prevalent and should be recognized as a major health concern in pediatrics internationally (1). The International Association for the Study of Pain (IASP) developed a classification of chronic pain diagnoses that distinguishes chronic primary pain and chronic secondary pain syndromes (2). Different from chronic secondary pain syndromes that are linked to an underlying condition (2), chronic primary pain cannot be explained by organic pathology (3). The most common pediatric primary chronic pain diagnoses include chronic headaches, chronic abdominal pain, chronic musculoskeletal and/or joint pain, and chronic back pain (1). Complex Regional Pain Syndrome, Type 1 (CRPS type 1) is also frequently seen in pediatric chronic pain clinics (4) and can have a significant biopsychosocial impact on children and youth (5). Chronic pelvic pain is also thought to be common in adolescent females, however the exact prevalence is unknown (6).

Chronic pain disorders are under-diagnosed in children and adolescents (4), causing significant delays in receiving specialized treatment (7, 8). Such delays are often due to diagnostic uncertainty in the chronic pain population since there is minimal evidence to support the diagnosis of “medically unexplained” pain in children (9). Pediatricians may especially experience diagnostic uncertainty in this population and there has been low agreement among pediatricians regarding chronic pain etiology and diagnostic approaches (10). Diagnostic uncertainty may be related to the tendency to complicate the diagnostic process in the chronic pain population (11) and likely increases the occurrence of unnecessary diagnostic tests. Conversely, misdiagnosing secondary pain syndromes as primary chronic pain can be harmful. Understanding pain etiology is considered the most important criterion when accepting and triaging patients to chronic pain programs (12), which highlights the need to enhance the diagnostic process for patients with primary chronic pain diagnoses. The general diagnostic process is thought to be iterative, with the goal of reducing diagnostic uncertainty, narrowing down diagnostic possibilities, and developing a more precise and complete understanding of a patient’s health problem (13). By adequately addressing the diagnostic process for pediatric primary chronic pain diagnoses, diagnostic expectations may be clarified which may reduce the occurrence of unnecessary diagnostic tests, streamline the referral process and facilitate the decision to accept or redirect patients into interdisciplinary pediatric chronic pain programs based on diagnostic completeness.

The purpose of this study was to outline diagnostic expectations for common primary chronic pain diagnoses in the pediatric population from the perspectives of specialized pediatric chronic pain providers. Our primary objectives were to attain expert consensus on important significant clinical indicators (i.e., red flags/signs of organic pathology) that are important to assess for in patients referred to interdisciplinary pediatric chronic pain programs, as well as to identify what diagnostic investigations are important to complete for patients who do not have significant clinical indicators, prior to acceptance into interdisciplinary pediatric chronic pain programs. For this study, diagnoses were limited to (1) Complex Regional Pain Syndrome, Type 1 (CRPS type 1), (2) Chronic Headaches, (3) Chronic Musculoskeletal and/or Joint Pain, (4) Chronic Back Pain, (5) Chronic Abdominal Pain and (6) Chronic Pelvic Pain. Our secondary objectives were to identify common courses of action that chronic pain providers take when patients are referred to them with significant clinical indicators/red flags (e.g., re-directing the referral, denying the referral, etc.), as well as utilization of Clinical Decision Support (CDS) tools and Patient Reported Outcome Measures (PROMs) that inform the decision to accept patients based on appropriateness.

## Materials and methods

### Design

We conducted a modified Delphi study, a well-recognized method for assessing expert opinion (14, 15), with pediatric chronic pain physicians, nurse practitioners and clinical nurse specialists. Our methodology was not considered a “classical Delphi”, which usually starts with an open-ended set of questions from participants (14). This approach has been critiqued to produce large amounts of questions that may not be well phrased which challenges the reliability and validity of the data and risks significant participant withdrawal (14). Instead, we conducted a literature search of relevant diagnostics and significant clinical indicators for each pain diagnosis lending to a more streamlined and evidence-based set of questions. We administered two-rounds of online surveys to develop consensus on the items that should be evaluated in the diagnostic investigation for the six pediatric primary chronic pain diagnoses listed above. Items included: (1) significant clinical indicators (i.e., red flags/signs of organic pathology); and in the absence of significant clinical indicators/ red flags: (2) necessary laboratory investigations; (3) necessary diagnostic imaging investigations; and (4) necessary diagnostic procedure investigations. We also assessed experts’ course of action if patients were referred with significant clinical indicators, as well as any CDS tools and PROMS they use in clarifying diagnoses and/ or facilitating their decision to accept patients into their programs.

### Expert participant panel

Participants were eligible based on their role (pediatric chronic pain physicians, nurse practitioners, clinical nurse specialists) and experience working in an interdisciplinary pediatric chronic pain program (current or past). It is suggested that the quality of information obtained by the Delphi technique is improved with numbers up to 13 participants (16). Therefore, the recruitment goal for this study was to have a minimum of 20 experts participate in the first round to account for attrition.

### Study procedures

**Figure 1:**
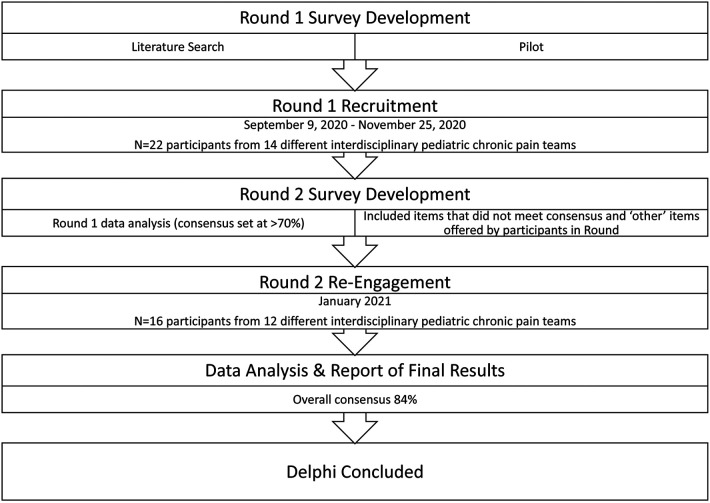
Delphi process flowchart.

This study was approved by the Research Ethics Boards at both the University of Ottawa (REB #H-11-19-5122) and the Children’s Hospital of Eastern Ontario (REB #2020058). Our procedures, analysis and reporting of results was guided by *The Delphi Technique in Nursing and Health Research Handbook* (14) and align with the *Guidance on Conducting and Reporting Delphi Studies (CREDES)* recommendations (17). Pediatric chronic pain experts were invited to participate in this study through the Pediatric Pain List Serve, which is an international internet forum maintained by Dalhousie University in Halifax, Nova Scotia. Recruitment included snowball sampling as many interested participants shared the survey invitation with eligible colleagues. Interested participants were asked to contact the Principal Investigator to confirm their interest and ensure eligibility. Confirmed and eligible participants were then sent an online link (*via* RedCap) to complete the first Delphi survey. Informed consent was obtained after participants read through the study’s Letter of Information, which stated “consent will be assumed upon completion of the questionnaire”. Therefore, for both Delphi rounds, consent was assumed following completion of both questionnaires. Following analysis of the first-round survey, respondents were contacted individually *via* email to invite them to participate in the second-round survey. For both rounds, reminder emails were sent to non-responders weekly for up to three weeks. Please see Figure 1 for an outline of our Delphi process.

### Survey development

Both surveys were organized based on the objectives listed above. Items were generated largely on available diagnostic literature of the six chosen pediatric primary chronic pain diagnoses, which was searched primarily through two point of care databases (*DynaMed Plus* and *RxTx*), that update clinical information frequently. Some diagnoses were not listed in either database, therefore supplemental articles were used to capture diagnostic information for the surveys. For purposes of clinical validity, the first-round survey was piloted with two pediatric chronic pain physicians and one pediatric pain nurse practitioner, which resulted in additional items added based on their recommendations. Individuals who participated in the pilot survey were not study participants and their results were not included in our analysis. The first-round survey included a total of 148 diagnostic items across the six pain diagnoses (CRPS type 1, *n* = 14; Chronic Headaches, *n* = 23; Chronic Musculoskeletal and/or Joint Pain, *n* = 30; Chronic Back Pain, *n* = 21; Chronic Abdominal Pain, *n* = 34; Chronic Pelvic Pain, *n* = 26). The second-round survey involved 85 diagnostic items involving the original items that did not reach consensus in round-one, as well as “other” items considered important by participants (CRPS type 1, *n* = 6; Chronic Headaches, *n* = 14; Chronic Musculoskeletal and/or Joint Pain, *n* = 20; Chronic Back Pain, *n* = 10; Chronic Abdominal Pain, *n* = 26; Chronic Pelvic Pain, *n* = 9). Questions regarding course of action for patients referred with significant clinical indicators were formatted as multiple choice and were not included in consensus. Participants also listed utilized CDS tools and PROMS and added additional feedback and comments as open-ended free text. The first-round and second-round surveys can be found in Supplementary Files S1, S2, respectively.

### Data collection and analysis

Participants were asked to rate their perceived degree of importance of significant clinical indicators/red flags and diagnostic items in the diagnostic investigation for each of the six chronic pain diagnoses on a 5-point Likert scale from 1 (not at all important) to 5 (extremely important). Since there is no standard threshold for defining consensus (i.e., recommendations have been found to be between 51% to 80%) (16), our analysis strategy was modeled after a Delphi study that evaluated expert consensus with the goal of developing a classification system for patients with low back pain (18). Responses with a rating of three or greater were considered important, while those with a rating of one or two were considered not important. To achieve group consensus in deeming an item important to consider or include in a referred patient, 70% or more of participants rated the item as important-extremely important. To achieve group consensus in deeming an item as not important to consider or include in a referred patient, 70% or more of participants rated the item as not at all important to somewhat important. Participants were also invited to offer “other” items they believed were important to include. The second-round survey involved the items that had not reached consensus in the first round, as well as the “other” items offered by participants. Participants were able to change their responses from the first-round survey based on the outlined results and were blinded to the identity of other participants to reduce response bias. A third round was not conducted since the overall degree of consensus met in Round 2 was high at 84%. Descriptive statistics including frequencies, percentages and medians and were used to describe responses across all participants, since the median is considered the optimal statistic for describing group agreement (15). Qualitative data was captured through additional feedback offered by participants and were analyzed by thematic analysis using an inductive approach (19). Data was coded by one author and all analytical decisions were validated by all other authors. There was general agreement from all authors.

## Results

### Expert demographics

The first-round survey included a total of 22 pediatric chronic pain experts from 14 different interdisciplinary teams and 4 different countries. Two participants indicated that they previously worked in an interdisciplinary team, however both had 10–20 years of working experience with the pediatric chronic pain population and were therefore still included in the study. Sixteen (72%) of those participants, from 12 different interdisciplinary teams and 4 different countries participated in the second-round survey. Reasons for withdrawal were not provided by participants. A summary of participant demographics can be found in Table 1.

**Table 1:** Participant demographics.

Item		Number of participants		Number of interdisciplinary teams	
		Round 1 *n* = 22 (%)	Round 2 *n* = 16 (%)	Round 1 (*n* = 14)	Round 2 (*n* = 12)
Geographical area of work	United States of America	11 (50)	9 (56)	7	6
	Canada	9 (41)	5 (31)	5	4
	France	1 (4.5)	1 (6)	1	1
	Australia	1 (4.5)	1 (6)	1	1
Clinical designation	Anesthesiologist	7 (32)	4 (25)		
	Pediatrician	4 (18)	2 (12.5)		
	Pediatric Rheumatologist	1 (4.5)	1 (6)		
	Nurse practitioner	6 (27)	6 (37.5)		
	Clinical nurse Specialist	4 (18)	3 (19)		
Level of education	Doctor of medicine	12 (54.5)	–		
	Master’s degree	8 (36)	–		
	Doctoral degree	1 (4.5)	–		
	Bachelor’s degree	1 (4.5)	–		
Years of working experience	0–5 years	3 (14)	3 (19)		
	5–10 years	6 (27)	4 (25)		
	10–20 years	9 (41)	8 (50)		
	20–30 years	2 (9)	1 (6)		
	30 + years	2 (9)	0		

### Degree of consensus on diagnostic items

Across both rounds, 84% (157/187) of both original and “other” diagnostic items reached consensus. The highest level of overall agreement was with CRPS type 1, followed by chronic headaches, chronic musculoskeletal and/or joint pain, chronic back pain, chronic abdominal pain, and chronic pelvic pain. Included in Table 2 lists the degree of consensus reached per pain location/diagnosis and domain, as well as the items that reached consensus and their deemed importance. Items that did not reach consensus are also listed in Table 2. Course of action participants take for referred patients with significant clinical indicators/red flags are listed in Table 3 and CDS tools used by participants to inform diagnoses are listed in Table 4.

**Table 2:** Consensus.

Degree of consensus by pain location/diagnosis	Degree of consensus by domain	Item	Original Item	“Other” Item added by Participant	Consensus Met	Decision
Complex regional pain syndrome (CRPS), Type 1 18/18 items (100%)	Significant Clinical Indicators (i.e., Clinical Red Flags/Signs of Organic Pathology) 7/7 items (100%)	Suspicion of active bone and/or soft tissue damage	•		√	Important
		Suspicion of neuropathies	•		√	Important
		Fever and/or chills	•		√	Important
		Neurovascular changes		•	√	Important
		History of pulselessness		•	√	Important
		History of trauma		•	√	Important
		History of surgery		•	√	Important
	Laboratory Investigations Required for Patients WITHOUT Significant Clinical Indicators 4/4 items (100%)	Erythrocyte Sedimentation	•		√	Not Important
		Serum C-Reactive Protein	•		√	Not Important
		Serum CBC	•		√	Not Important
		Serum Creatinine Kinase	•		√	Not Important
	Diagnostic Imaging Investigations Required for Patients WITHOUT Significant Clinical Indicators 5/5 items (100%)	Bone Scan of affected area(s)	•		√	Not Important
		X-Ray of affected area(s)	•		√	Not Important
		Magnetic Resonance Imaging of affected area(s)	•		√	Not Important
		Duplex Ultrasonography of affected area(s)	•		√	Not Important
		Computed Tomography	•		√	Not Important
	Diagnostic Procedures Required for Patients WITHOUT Significant Clinical Indicators 2/2 items (100%)	Local Anesthetic Injection Trial	•		√	Not Important
		Nerve Conduction Studies	•		√	Not Important
Chronic headaches 32/35 items (91%)	Significant Clinical Indicators (i.e., Clinical Red Flags/Signs of Organic Pathology) 17/18 items (94%)	Neurological abnormalities	•		√	Important
		Child is between 3 and 5 years	•		√	Important
		Systematic signs and symptoms	•		√	Important
		Headache of sudden onset	•		√	Important
		Headache wakes from sleep	•		√	Important
		Described by patient as “worst headache of life”	•		√	Important
		New or different severe headache	•		√	Important
		Headache that worsens with Valsalva	•		√	Important
		Change in headache frequency	•		√	Important
		Vomiting		•	√	Important
		Family history of neurological disease		•	√	Important
		History of cancer		•	√	Important
		History of ventriculoperitoneal (VP) shunt		•	√	Important
		Postural headache		•	√	Important
		Headache upon wakening		•	√	Important
		Loss of developmental milestones		•	√	Important
		Weight loss/loss of appetite		•	√	Important
		History of tooth pain		•	X	*Did not reach consensus*
	Laboratory Investigations Required for Patients WITHOUT Significant Clinical Indicators 9/10 items (90%)	Serum Electrolytes	•		√	Not Important
		Serum Glucose	•		√	Not Important
		Serum Albumin	•		√	Not Important
		Serum Complete Blood Cell Count	•		√	Not Important
		Serum Blood Urea Nitrogen	•		√	Not Important
		Serum Calcium	•		√	Not Important
		Serum Creatinine	•		√	Not Important
		Serum Vitamin D		•	√	Not Important
		Serum Ferritin		•	√	Not Important
		Serum Thyroid Function	•		X	*Did not reach consensus*
	Diagnostic Imaging Investigations Required for Patients WITHOUT Significant Clinical Indicators 2/2 items (100%)	Magnetic Resonance Imaging	•		√	Not Important
		Computed Tomography	•		√	Not Important
	Diagnostic Procedures Required for Patients WITHOUT Significant Clinical Indicators 4/5 items (80%)	Temporomandibular Joint (TMJ) Assessment	•		√	Not Important
		Lumbar puncture	•		√	Not Important
		Sleep study	•		√	Not Important
		Papilledema assessment		•	√	Not Important
		Visual acuity exam		•	X	*Did not reach consensus*
Chronic musculoskeletal and/or joint pain 35/39 items (90%)	Significant Clinical Indicators (i.e., Clinical Red Flags/Signs of Organic Pathology) 11/13 items (85%)	Unexplained weight loss	•		√	Important
		Systematic signs and symptoms	•		√	Important
		Pain and stiffness in the morning	•		√	Important
		Arthralgia with redness and edema	•		√	Important
		History of significant physical trauma	•		√	Important
		Radiculopathy	•		√	Important
		Bony tenderness	•		√	Important
		History of congenital anomalies	•		√	Important
		Pain at night	•		√	Important
		History of cancer		•	√	Important
		Positive trigger points		•	√	Important
		History of prior surgeries	•		X	*Did not reach consensus*
		Known Ehler’s- Danlos Syndrome (EDS)		•	X	*Did not reach consensus*
	Laboratory Investigations Required for Patients WITHOUT Significant Clinical Indicators 18/19 items (95%)	Serum Creatinine Kinase	•		√	Not Important
		Serum C-Reactive Protein	•		√	Not Important
		Serum Complete Blood Cell Count	•		√	Not Important
		Serum Thyroid Function	•		√	Not Important
		Serum Tissue Transglutaminase	•		√	Not Important
		Serum Antinuclear Antibodies	•		√	Not Important
		Serum Rheumatoid Factor	•		√	Not Important
		Serum Calcium	•		√	Not Important
		Serum Blood Urea Nitrogen	•		√	Not Important
		Serum Albumin	•		√	Not Important
		Serum Glucose	•		√	Not Important
		Serum Creatinine	•		√	Not Important
		HLA B27		•	√	Not Important
		Vitamin D level		•	√	Not Important
		Vitamin B12 level		•	√	Not Important
		Folate level		•	√	Not Important
		Complement level		•	√	Not Important
		Urinalysis		•	√	Not Important
		Serum Erythrocyte Sedimentation Rate (ESR)	•		X	*Did not reach consensus*
	Diagnostic Imaging Required for Patients WITHOUT Significant Clinical Indicators 3/4 items (75%)	Ultrasound of affected area(s)	•		√	Not Important
		Magnetic Resonance Imaging of affected area(s)	•		√	Not Important
		Computed Tomography of affected area(s)	•		√	Not Important
		X-Ray of affected area(s)	•		X	*Did not reach consensus*
	Diagnostic Procedures Required for Patients WITHOUT Significant Clinical Indicators 3/3 items (100%)	Muscle biopsy	•		√	Not important
		Nerve Conduction Studies	•		√	Not important
		Electromyography	•		√	Not important
Chronic back pain 19/24 items (79%)	Significant Clinical Indicators (i.e., Clinical Red Flags/Signs of Organic Pathology) 12/14 items (86%)	Incontinence (bladder and/or bowel)	•		√	Important
		Unexplained weight loss	•		√	Important
		Fever and chills	•		√	Important
		History of cancer	•		√	Important
		Widespread neurological symptoms	•		√	Important
		History of immunocompromised condition	•		√	Important
		History of infection or trauma	•		√	Important
		Bilateral sciatica	•		√	Important
		Radiculopathy	•		√	Important
		Unrelenting night pain	•		√	Important
		Pain unrelated to activity	•		√	Important
		Redness/edema of painful site		•	√	Important
		Constant pain		•	X	*Did not reach consensus*
		History of scoliosis		•	X	*Did not reach consensus*
	Laboratory Investigations Required for Patients WITHOUT Significant Clinical Indicators 5/6 items (83%)	Serum CBC	•		√	Not Important
		Serum Erythrocyte Sedimentation Rate	•		√	Not Important
		Serum Calcium	•		√	Not important
		Serum Alkaline Phosphate	•		√	Not important
		Antinuclear Antibody (ANA) level		•	√	Not Important
		Serum C-Reactive Protein	•		X	*Did not reach consensus*
	Diagnostic Imaging Investigations Required for Patients WITHOUT Significant Clinical Indicators 2/4 items (50%)	Computed Tomography of affected area(s)	•		√	Not important
		Ultrasound of affected area(s)	•		√	Not important
		X-Ray of Affected Area(s)	•		X	*Did not reach consensus*
		Magnetic Resonance Imaging (MRI) of Affected Area(s)	•		X	*Did not reach consensus*
Chronic abdominal pain 34/43 items (79%)	Significant Clinical Indicators (i.e., Clinical Red Flags/Signs of Organic Pathology) 13/14 items (93%)	Bloody emesis	•		√	Important
		Bloody stools	•		√	Important
		Concern or diagnosis of an eating disorder	•		√	Important
		Unexplained weight loss	•		√	Important
		Systemic signs and symptoms	•		√	Important
		Persistent vomiting	•		√	Important
		Persistent diarrhea	•		√	Important
		History of prior surgeries	•		√	Important
		Persistent RUQ/RLQ pain	•		√	Important
		History of trauma		•	√	Important
		Referred back pain		•	√	Important
		Bilious emesis		•	√	Important
		Pain that wakes from sleep		•	√	Important
		Family history of gastrointestinal cancer		•	X	*Did not reach consensus*
	Laboratory Investigations Required for Patients WITHOUT Significant Clinical Indicators 12/20 items (60%)	Serum C-Reactive Protein	•		√	Not Important
		Serum Albumin	•		√	Not Important
		Serum Creatinine	•		√	Not Important
		Serum Blood Urea Nitrogen	•		√	Not Important
		Serum Thyroid Function	•		√	Not Important
		Fecal Culture / Sensitivity	•		√	Not Important
		Fecal Ova / Parasite	•		√	Not Important
		Urinalysis	•		√	Not Important
		Serum Calcium	•		√	Not Important
		Urine Culture / Sensitivity	•		√	Not Important
		Fecal Occult Blood Test	•		√	Not Important
		Serum Erythrocyte Sedimentation Rate		•	√	Not Important
		Serum Complete Blood Cell Count (CBC)	•		X	*Did not reach consensus*
		Serum Electrolytes	•		X	*Did not reach consensus*
		Serum Liver Function	•		X	*Did not reach consensus*
		Serum Glucose	•		X	*Did not reach consensus*
		Serum Lipase/Amylase	•		X	*Did not reach consensus*
		Serum Tissue Transglutaminase (TTG)	•		X	*Did not reach consensus*
		H. Pylori screen		•	X	*Did not reach consensus*
		Fecal calprotectin		•	X	*Did not reach consensus*
	Diagnostic Imaging Investigations Required for Patients WITHOUT Significant Clinical Indicators 4/4 items (100%)	Abdominal Ultrasound	•		√	Not Important
		Abdominal x-Ray	•		√	Not Important
		Abdominal Magnetic Resonance Imaging	•		√	Not Important
		Abdominal Computed Tomography	•		√	Not Important
	Diagnostic Procedures Required for Patients WITHOUT Significant Clinical Indicators 5/5 items (100%)	Endoscopy with biopsies	•		√	Not Important
		Endoscopy without biopsies	•		√	Not Important
		Hydrogen Breath Test (for fructose/lactose sensitivities)	•		√	Not Important
		Local anesthetic injection to rule in or out ACNES	•		√	Not Important
		Gastric emptying study		•	√	Not Important
Chronic pelvic pain 19/28 items (68%)	Significant Clinical Indicators (i.e., Clinical Red Flags/Signs of Organic Pathology) 12/14 items (86%)	History of sexual trauma	•		√	Important
		Excessive or unexplained weight loss	•		√	Important
		Pelvic mass	•		√	Important
		History of physical trauma	•		√	Important
		Tenesmus	•		√	Important
		Testicular mass	•		√	Important
		History of congenital anomalies	•		√	Important
		Vaginal discharge	•		√	Important
		Rectal bleeding	•		√	Important
		Post-coital bleeding	•		√	Important
		Dysmenorrhea	•		√	Important
		Menorrhagia	•		√	Important
		Dyspareunia		•	X	*Did not reach consensus*
		Hematuria		•	X	*Did not reach consensus*
	Laboratory Investigations Required for Patients WITHOUT Significant Clinical Indicators 0/5 items (0%)	Urinalysis	•		X	*Did not reach consensus*
		Urine Culture / Sensitivity	•		X	*Did not reach consensus*
		Sexual Transmitted Infection Swab	•		X	*Did not reach consensus*
		Serum/Urine Beta HcG	•		X	*Did not reach consensus*
		Serum Complete Blood Cell Count	•		X	*Did not reach consensus*
	Diagnostic Imaging Required for Patients WITHOUT Significant Clinical Indicators 3/5 items (60%)	Magnetic Resonance Imaging of Pelvis	•		√	Not important
		Transvaginal Ultrasound	•		√	Not Important
		Computed Tomography of Pelvis	•		√	Not important
		Testicular Ultrasound	•		X	*Did not reach consensus*
		Abdominal / Pelvis Ultrasound	•		X	*Did not reach consensus*
	Diagnostic Procedures Required for Patients WITHOUT Significant Clinical Indicators 4/4 items (100%)	Colonoscopy	•		√	Not important
		Diagnostic Laparoscopy	•		√	Not important
		Barium Enema	•		√	Not important
		Cystoscopy	•		√	Not important

Consensus threshold = to deem an item important to consider/include in a referred patient, >70% of participants must rate as important”- “extremely important”. To deem an item not important to consider/include in a referred patient, >70% of participants must rate as “not at all important”- “somewhat important”.

**Table 3:** Course of action if patient has significant clinical indicators (Red flags) prior to acceptance into interdisciplinary pediatric chronic pain program.

Type of pain	Deny patient with no suggestions to referring provider, *n* (%)	Deny patient with suggestions to referring provider, *n* (%)	Re-direct referral to emergency department, *n* (%)	Re-direct referral to specialty service, *n* (%)	Accept patient and request patient complete required work-up, *n* (%)	Accept patient and assess yourself, *n* (%)
Complex regional Pain syndrome type 1	1 (4.5)	1 (4.5)	2 (9)	8 (36)	2 (9)	6 (27)
Chronic headaches	0	2 (9)	8 (36)	4 (18)	3 (14)	4 (18)
Chronic musculoskeletal/ joint pain	1 (4.5)	2 (9)	0	11 (50)	3 (14)	3 (14)
Chronic back pain	0	2 (9)	4 (18)	9 (41)	1 (4.5)	3 (14)
Chronic abdominal pain	0	3 (14)	0	10 (45)	4 (18)	1 (5)
Chronic pelvic pain	1 (4.5)	2 (9)	0	12 (55)	4 (18)	0

**Table 4:** Use of clinical decision support tools in accepting patients to interdisciplinary pediatric chronic pain programs.

Type of pain	Yes *N (%)*	No *N (%)*	List of clinical decision support tools/diagnostic algorithms *(number of participants who mentioned tool)*
Complex regional pain syndrome type 1	15 (68)	7 (32)	• Budapest criteria (*n* = 14)
Chronic headaches	11 (50)	11 (50)	• The international classification of headache disorders (*n* = 10) • American academy of neurology and american headache society (*n* = 1) • PedMidas (*n* = 1)
Chronic musculoskeletal/joint pain	2 (9)	20 (91)	• Beighton score (*n* = 1)
Chronic back pain	1 (5)	21 (95)	• American academy of pediatrics recommendations (*n* = 1) • American academy of family practice guidelines (*n* = 1) • American college of rheumatology guidelines (*n* = 1)
Chronic abdominal pain	6 (27)	15 (68)	• Rome III criteria (*n* = 1) • American academy of pediatrics (*n* = 5)
Chronic pelvic pain	1 (5)	21 (95)	• American academy of family practice guidelines (*n* = 1)

### Complex regional pain syndrome, type 1

CRPS type 1 was the only diagnosis that met 100% consensus within all domains. All significant clinical indicators/red flags were deemed important to assess for, while all diagnostic investigations were considered not important to complete prior to referral for patients without significant clinical indicators/red flags. Some participants (*n* = 8, 36%) indicated that for patients referred with significant clinical indicators/red flags, they would re-direct the referral to a specialty service, while 27% (*n* = 6) would accept the patient and assess themselves. CDS use was reported to be the highest with CRPS type 1, with 15 of 22 participates reporting that they use a CDS tool, 14 of whom specified that they follow the *Budapest Criteria* (20).

### Chronic headaches

Consensus was reached on 91% (*n* = 32) of chronic headache diagnostic items, where most significant clinical indicators/red flags were deemed important (*n* = 17, 94%) to consider in referred patients. Most laboratory items (*n* = 9, 90%) were considered not important to conduct prior to referral in patients without significant clinical indicators/red flags, followed by 80% (*n* = 4) of diagnostic procedures and 100% (*n* = 2) of diagnostic imaging investigations. Some participants (*n* = 8, 36%) indicated that for patients referred with significant clinical indicators/red flags, they would re-direct them to an emergency department (*n* = 8, 36%). Half of the sample (*n* = 11, 50%) indicated that they use a CDS tool for chronic headache referrals, ten of whom specifically mentioned the *International Classification of Headache Disorders* (21).

### Chronic musculoskeletal and/or joint pain

Consensus was reached on 90% (*n* = 35) of chronic musculoskeletal and/or joint pain diagnostic items, where 85% (*n* = 11) of significant clinical indicators/red flags were deemed important to consider in referred patients. In terms of diagnostics, 95% (*n* = 18) of laboratory, 75% (*n* = 3) of diagnostic imaging investigations and all diagnostic procedure investigations (*n* = 3, 100%) were considered not important to conduct prior to referral in patients without significant clinical indicators/red flags. Half of participants (*n* = 11, 50%) indicated that they would redirect referred patients with significant clinical indicators/red flags to a speciality service. Few participants (*n* = 2) reported using a CDS tool, one of whom mentioned the *Beighton criteria* (22).

### Chronic back pain

Consensus was reached on 79% (*n* = 19) of chronic back pain diagnostic items, with 86% (*n* = 5) of significant clinical indicators/red flags deemed important to consider in referred patients. Most laboratory (*n* = 5, 83%) and half of diagnostic imaging (*n* = 2, 50%) investigations were considered not important to conduct prior to referral in patients without significant clinical indicators/red flags. A selection of participants (*n* = 9, 41%) specified that they would re-direct referred patients with significant clinical indicator/red flags to a specialty service, while only one participant reported using CDS tools for chronic back pain referrals, referencing the *American Academy of Pediatrics* (23), the *American Academy of Family Physicians* (24) and the *American College of Rheumatology* (25).

### Chronic abdominal pain

Consensus was reached on 79% of chronic abdominal pain items, with 93% (*n* = 13) of significant clinical indicators/red flags deemed important to consider in referred patients. All diagnostic imaging (*n* = 4, 100%) and diagnostic procedure (*n* = 5, 100%) investigations were considered not important to conduct prior to referral in patients without significant clinical indicators/red flags. A portion of laboratory investigations (*n* = 12, 60%) met consensus and were also considered not important. Nearly half of participants (*n* = 10, 45%) indicated that they would re-direct patients referred with significant clinical indicators/red flags to a specialty service, while six participants reported using a CDS tool for chronic abdominal pain referrals, five of whom referenced the *American Academy of Pediatrics* (23) and one reported following the *Rome III criteria* (26).

### Chronic pelvic pain

Consensus was reached on 68% (*n* = 19) of chronic pelvic pain items, with 86% (*n* = 12) of significant clinical indicators/red flags deemed important to consider for referred patients. All four diagnostic procedure investigations and three of five (60%) diagnostic imaging investigations were considered not important to conduct prior to referral in patients without significant clinical indicators/red flags. Consensus was not reached on any of the five laboratory investigations. Some participants (*n* = 12, 55%) specified that they would re-direct patients with significant clinical indicators/red flags to a speciality service and one participant reported using a CDS tool for chronic pelvic pain referrals, referencing the *American Academy of Family Physicians* (24).

### Patient reported outcome measures (PROMs)

The most frequently utilized PROMs that experts reported they prefer to have completed prior to referral are the: Patient Reported Outcome Measurement Information System (PROMIS) (27) (*n* = 7/22 participants; 16/14 teams), Functional Disability Inventory (FDI) (28) (*n* = 7/22 participants; 5/14 teams), Numerical Rating Scale (NRS) (29) (*n* = 6/22 participants; 4/14 teams), Pain Catastrophizing Scale (PCS) (30) (*n* = 4/22 participants; 3/14 teams), Pediatric Quality of Life Inventory (PedsQL) (31) (*n* = 2/22 participants; 2/14 teams), Faces Pain Scale Revised (FPS, R) (32) (*n* = 2/22 participants; 2/14 teams), Child Activity Limitations Interview (CALI) (33) (*n* = 2/22 participants; 2/14 teams). Only single participants from different teams mentioned each of the following PROMs: Self Determination Scale (34), Insomnia Severity Index (35), Pain Related Cognition Questionnaire for Children (PRCQ-C) (36), Childhood Sleep Habits Questionnaire (37), Patient Health Questionnaire-9 (PHQ-9) (38), CRAFT Substance Use Screening Tool (39), Symptom Severity Score (40), Children’s Depression Inventory (CDI) (41), Body Map (42), BATH Adolescent Pain Questionnaire (43) and the Brief Pain Inventory (BPI) (44).

### Supporting data

Participant feedback related to the contextual influences that impact their decision to accept or re-direct referred patients based on diagnostic completeness was grouped into four categories: (1) chronic pain program contexts, (2) diagnostic role, (3) quality of referral data, and (4) evidence-informed decision making. This data highlights that although there is variation in chronic pain models and philosophies, diagnostic completeness is considered important before accepting patients into chronic pain programs. Furthermore, the quality and quantity of referral data impacts how triage decisions are made after patients are accepted (i.e., how they are prioritized). It was also noted across this group of experts that it is typically not their role to complete diagnostic investigations, but rather to collaborate with referring providers and other speciality services who take responsibility for selecting and conducting diagnostic investigations. The lack of evidence-based guidance to inform the diagnostic process with pediatric chronic pain patients was highlighted, and there is participant interest in using CDS tools and PROMS to support the decision to accept or re-direct patients based on their diagnostic completeness and appropriateness. A summary of details and exemplar quotes can be found in Table 5.

**Table 5:** Supporting qualitative data.

Topic	Theme	Frequency	Belief statement	Frequency	Exemplar quote
Context of chronic pain programs	Clinic models and philosophies	4	Chronic pain program models and philosophies vary	1	*The diagnostic approach is somewhat dependent on the clinic model/philosophy. There is a spectrum of clinic models from full diagnostic clinics to consultative only (**Nurse Practitioner**)*
			Adequate completion of investigations is required prior to accepting patients into chronic pain programs	1	*In general - our clinic does not accept any patients who have not been adequately investigated for an acute medical illness that might explain the pain (**Pediatrician**)*
			Screening patient data prior to accepting them into a chronic pain program is important	1	*A thorough screening is done prior to admitting into program. At times we will do a consultation and if needed then refer. If there is significant past trauma, there are times we will refer to tx then they can return to program if needed (**Nurse Practitioner**)*
			Trusting in specialized colleagues is important when deeming work up complete	1	*We also trust that our specialty colleagues have more expertise in assessing than we do. For example - if rheumatology orders no tests for a kid with widespread pain - that is fine. They are the experts in assessing these symptoms. If GI does no investigations for a kid with abdo pain that is okay with us. So, it isn’t really about assessing what prior test have been done - it is about WHO has seen the kid and who has made the referral to us (**Pediatrician**)*
Diagnostic role	Chronic pain clinician does not assume diagnostic role	21	Work in parallel collaboration with specialized provider	9	*Assess patient and then refer to appropriate specialty (**Anesthesiologist**)*
			Accept patient only after being seen by relevant specialist	4	*We only accept headache referrals if the patient has been assessed by a child neurologist. So even if these signs are present, we will accept the referral, assuming that the neurologist has assessed and done the necessary investigations (**Pediatrician**)*
			Work in collaboration with Primary Care Provider	3	*If workup is not done, we would recommend the referral source arrange for this - but we do not redirect the referral. That is the family docs role (not ours) (**Pediatrician**)*
			Accept patient but defer assessment until work up complete	2	*It is not uncommon to accept a patient but defer until further work up complete (**Nurse Practitioner**)*
			Re-direct patient to ER to facilitate further work-up	2	*In our practice, going to ED is a way to facilitate getting the work up rather than waiting for insurance (**Nurse Practitioner**)*
			Do not accept patient until work-up is complete	1	*We would only accept a patient who has had the necessary work-up for abdo pain (**Pediatrician**)*
	Chronic pain clinician partially assumes diagnostic role	1	Depending on case, start work-up within chronic pain service	1	*If concern for disordered eating, would refer patient to adolescent medicine. If primary concern is GI pathology would start work-up myself and refer to GI for evaluation (**Pediatric Rheumatologist**)*
Quality of referral data	When referral data is lacking	1	It is common to receive referrals that do not have adequate information to inform a triage decision	1	*We are generally just happy to receive patient records when we receive a referral, much less specifically documented criteria. While common among pain professionals, these types of evaluations are difficult to get from community providers. Even a numeric pain scale seems like a challenge (**Nurse Practitioner**)*
	When referral data is optimal	2	When the work up is complete, triage decisions are easier to make	2	*Usually by the time we get them, the work up is complete- which makes it easy (**Nurse Practitioner**)*
Evidence informed decision making	CDS utilization	1	We do not use CDS tools but are interested in using them	1	*We do not use any decision support tools, but we’d love to know more about them if there are any validated for kids (**Anesthesiologist**)*
	PROM utilization	2	Preference to have PROMs completed prior to first chronic pain assessment appointment	1	*Patient reported outcomes inform the diagnosis in the initial assessment. We would accept the referral but strongly recommend completion of these outcomes prior to their first appointment (**Clinical Nurse Specialist**)*
			Availability of standardized data sets	1	*In Australia we have a national data set for referrals ePPOC this includes: FPS-R, Body map, PedsQL, FDI and over 13’s complete pain related worries from BathAPQ (**Clinical Nurse Specialist**)*
	Lack of evidence-based guidance	2	There is a lack of evidence-based guidance to inform diagnostic approach with pediatric chronic pain patients	2	*Unfortunately, there are no pediatric specific diagnostic approaches for chronic pain syndromes. This is a need that would provide us with a standard for which to diagnose and allow other specialists to refer to pain physicians sooner if there were better discriminating tool (**Anesthesiologist**)*

## Discussion

This study identified 72 significant clinical indicators/red flags that were deemed important to assess for in the diagnostic investigation of six pediatric primary chronic pain diagnoses, as well as 85 diagnostic investigations that were considered not important to complete prior to chronic pain referral in the absence of significant clinical indicators/red flags. Although classification of these items may help to reduce diagnostic uncertainty and clarify diagnostic expectations from the perspectives of specialized pediatric chronic pain providers, it is prudent to recognize that additional research is needed to attain further consensus amongst common referring providers and other specialty services.

There was good consensus to support a general recommendation to not conduct diagnostic investigations in the absence of significant clinical indicators/red flags prior to referral. This is in line with the general recommendations included in the CDS tools reported by participants. Despite this, there remains a delay between the onset of pain and the time that specialized care is received (7, 8). A recent prospective study investigating wait times for youth referred to interdisciplinary pediatric chronic pain programs found the average wait time to be 197.5 days, which caused increased anxiety and frustration for patients and families (44). Although reasons for long wait times were not examined, authors from that study emphasized the need to investigate referral practices of pediatric interdisciplinary chronic pain programs (44). One possible factor that increases wait times may be the prolonged extent of diagnostic investigations conducted on chronic pain patients prior to referral. A recent systematic review examining the magnitude and nature of inappropriately used clinical practices in Canada in all health sectors revealed that approximately 47% of diagnostic tests are over-used (45). Many practitioners order numerous diagnostic tests with chronic pain patients in fear of missing an organic cause to patients’ pain, and in hopes of providing reassurance to patients and families (46). Interestingly, a qualitative study exploring the perception of diagnostic uncertainty in youth with chronic pain demonstrated that even if diagnostic tests were negative, they did not provide relief to families (47). It is possible that many unnecessary diagnostic tests are being ordered due to the range of ambiguous symptomatology reported by parents of children with chronic pain (48, 49), which may cloud the diagnostic picture and lead to treatment delays. A recent qualitative study investigating the diagnostic uncertainty of pediatricians evaluating chronic pain patients suggests that the decision to stop diagnostic testing on patients with unexplained chronic pain is ambiguous, complicated, and determined by many patient and physician factors (50). Further complicating this decision includes patient and family readiness to accept their chronic pain diagnosis, since 40% of parents of youth referred to pediatric chronic pain programs do not (51). Instead, these families are described as “relentlessly” searching for an alternative diagnosis they believe has been missed by their physician (51). This creates a unique circumstance for the referring provider who is attempting to juggle resource utilization and patient expectations, all the while ensuring secondary causes for pain have been ruled out.

Qualitative findings from this study highlight that many chronic pain providers do not assume a diagnostic role and that the quantity and quality of referral data is generally lacking. This presents a noteworthy gap between the expectations of referring providers and chronic pain providers who accept patients into chronic pain programs. Such challenges have potential to lead to inconsistent and complicated diagnostic processes that can influence referral practices and the decision to accept or re-direct a patient from a chronic pain program, which further worsens wait time to receiving specialized care.

### Implications for future research

The identified list of significant clinical indicators/red flags and diagnostic investigations that are required prior to referral to interdisciplinary pediatric chronic pain programs will be helpful to include in the development of a series of CDS tools aimed to clarify diagnostic expectations and guide the decision to accept or re-direct patients with primary chronic pain diagnoses into interdisciplinary pediatric chronic pain programs based on diagnostic completeness. Our next cumulative steps will be to: (1) conduct a qualitative study exploring the decision-making practices of pediatric chronic pain nurses who accept and triage patients into their programs, and then (2) implement a user-centered design study with a team of referring providers and pediatric chronic pain providers to identify relevant items and acceptable processes that will expand to the development of clinically useful CDS triage tools for primary chronic pain diagnoses. Although it was not an objective of this study, future exploration should consider the influence that mental health symptoms have on chronic primary pain diagnoses from the perspectives of psychologists and other mental health providers, since it is considered a significant co-morbidity (52). We hope our study can be expanded in the future to include more participants from countries outside of North America to capture a more global view of diagnostic expectations for interdisciplinary pediatric chronic pain programs across the world.

### Strengths

To our knowledge, this is the first study of its kind to attain expert consensus on a large list of significant clinical indicators/red flags and required diagnostic investigations for six common pediatric primary chronic pain diagnoses from the perspectives of pediatric chronic pain experts. The diversity of respondents who were willing to participate in this study highlights an international and inter-role interest in the topic of diagnostic clarity for children and adolescents with primary chronic pain disorders. Justification for this study was qualitatively validated by participants, which highlights its relevancy to the population and community of chronic pain providers.

### Limitations

It is important to acknowledge the influence of bias on the validity and reliability of the Delphi method. Because this method relies on judgements, variances of results can be influenced by situation and personal bias (13, 16). Only 16 of the 22 experts participated in the second-round survey, challenging the generalizability of results. Further to this, most participants were from North America and thus findings are not geographically diverse. There is an element of selection bias, since only those subscribed to the Pain List Serve were recruited. Furthermore, this study was limited to two rounds. Although a high degree of overall consensus was met, conducting a third round may have influenced overarching results, particularly regarding chronic pelvic pain which demonstrated lowest degree of consensus. It is important to mention that the additional feedback offered by participants, identified as qualitative data, was not guided by traditional qualitative methodology, and therefore results should be interpreted with caution.

## Conclusion

There is general agreement amongst pediatric chronic pain experts in this study regarding diagnostic expectations for patients referred to interdisciplinary chronic pain programs for six common primary chronic pain diagnoses. There is also a universal consensus not to require diagnostic tests prior to acceptance into such programs for patients without significant clinical indicators or red flags. Despite this, the literature points to significant delays in receiving specialized treatment, which amongst many other potential factors such as capacity and resource limitations, may be related to conducting unnecessary diagnostic tests and over complicating the diagnostic process. Items that met consensus in this study may help to clarify diagnostic expectations for patients with primary chronic pain diagnoses referred to interdisciplinary chronic pain programs. As a next step, it will be crucial to include the perspectives of referring providers and other relevant speciality services since they commonly assume the diagnostic role. Findings from this study, combined with our planned future work, will result in the development of a series of user-centered, evidence-based, and clinically useful CDS triage tools for interdisciplinary pediatric chronic pain programs. We believe this has potential to ease the diagnostic process for referring providers, enhance the decision to accept or re-direct referred patients based on their diagnostic completeness and streamline the pathway to accessing specialized chronic pain evaluation and treatment. We hope this can ultimately reduce the burden of chronic pain on patients, their families, and the healthcare system.

## Data Availability

The raw data supporting the conclusions of this article will be made available by the authors, without undue reservation.
