# Supplementary material for: Attaining expert consensus on diagnostic expectations of primary chronic pain diagnoses for patients referred to interdisciplinary pediatric chronic pain programs: A delphi study with pediatric chronic pain physicians and advanced practice nurses
Source: Front Pain Res (Lausanne). 2022 Sep 12;3:1001028. doi: 10.3389/fpain.2022.1001028 (PMC9632850; doi:10.3389/fpain.2022.1001028)
Supplement: Supplementary file 1 [file Datasheet1.pdf]

Confidential

Delphi Survey: Attaining Expert Consensus on Significant Clinical Indicators and Diagnostic Approaches that are Required on Patients Prior to Acceptance into Interdisciplinary Pediatric Chronic Pain Programs

You are invited to participate as an expert in the field of pediatric chronic pain in the following Delphi survey conducted by the Principle Investigator. The purpose of using a Delphi technique is to assess group consensus about a topic in their respective field of work. This study is part of a larger mixed methods study intended to create a Clinical Decision Support (CDS) tool for nurses triaging patients to interdisciplinary pediatric chronic pain programs.

Purpose of the Study: The purpose of this study is to attain expert consensus on all necessary medical diagnostic approaches and indicators that are required on patients prior to acceptance into interdisciplinary pediatric chronic pain programs for common primary chronic pain diagnoses in the pediatric population. For the purpose of this study, diagnoses will be limited to 1) Chronic headaches, 2) Chronic abdominal pain, 3) Chronic pelvic pain, 4) Chronic musculoskeletal and joint pain, 5) Chronic back pain and 6) Complex Regional Pain Syndrome (CRPS) Type 1.

Specific study objectives are to attain expert consensus on the following for the common pediatric chronic primary pain diagnoses listed above:

1. Confirm list of significant clinical indicators (i.e., clinical red flags/ clinical signs of organic pathology) for each diagnosis

2. Identify best course of action if referred patient has significant clinical indicators for each diagnosis

3. Identify most appropriate lab work if patient has no significant clinical indicators for each diagnosis

4. Identify most appropriate diagnostic imaging if patient has no significant clinical indicators for each diagnosis

5. Identify most appropriate diagnostic procedures if patient has no significant clinical indicators for each diagnosis

6. Identify suggested diagnostic algorithms and/ or guidelines and/ or criteria to identify necessary diagnostic investigations and indicators for each diagnosis respectively

Please complete the survey below.

Thank you!

Do you work with the pediatric chronic pain population?	◯ Yes<br>◯ No
What is your medical or nursing designation?	◯ Anesthesiologist<br>◯ General Practitioner<br>◯ Paediatrician<br>◯ Registered Nurse<br>◯ Nurse Practitioner<br>◯ Clinical Nurse Specialist<br>◯ Advanced Practice Nurse<br>◯ Other
If your medical or nursing designation is not listed above, please provide it here.	______________________________
What is your highest level of education?	◯ Bachelor's Degree<br>◯ Master's Degree<br>◯ Doctorate Degree<br>◯ Doctor of Medicine Degree

Please list any other diplomas/ degrees/ certifications you have achieved that are not listed in the question above.	__________________________________
Do you work in an interdisciplinary pediatric chronic pain program/ clinic/ team? (*To note - we define 'interdisciplinary' as a combination of two or more clinical disciplines that work together within one team to serve the pediatric chronic pain population. Disciplines may include, but are not limited to: physicians, nurses, physiotherapists, psychologists, psychiatrists, occupational therapists, pharmacists, recreational therapists, etc.)	◯ Yes<br>◯ No
Please state the clinical role you have within your team/ clinic/ program	____________________________
How many years of experience do you have working with the pediatric chronic pain population?	◯ 0-5 years<br>◯ 5-10 years<br>◯ 10-20 years<br>◯ 20-30 years<br>◯ 30+ years
In what geographical location do you work?	◯ Canada<br>◯ United States of America<br>◯ Europe<br>◯ Australia<br>◯ New Zealand<br>◯ South America<br>◯ Asia<br>◯ Africa
Please indicate the province/ state within which you work.	____________________________

Please rate the importance of considering the following significant clinical indicators (i.e., clinical red flags/ clinical signs of organic pathology) in the diagnostic work-up of referred patients with chronic headaches.

	Not at all important	Somewhat important	Important	Very important	Extremely important
Child is between 3 and 5 years of age	◯	◯	◯	◯	◯
New or different severe headache	◯	◯	◯	◯	◯
Change in headache frequency	◯	◯	◯	◯	◯
Described by patient as 'worst headache of life'	◯	◯	◯	◯	◯
Headache wakes from sleep	◯	◯	◯	◯	◯
Headache of sudden onset	◯	◯	◯	◯	◯

Headache that worsens with Valsalva (i.e., coughing, sneezing, straining)	◯	◯	◯	◯	◯
Neurological abnormalities (i.e., history of seizures, weakness, altered level of consciousness, papilledema, etc.)	◯	◯	◯	◯	◯
Systemic signs and symptoms (i.e., fever, weight loss, rash, joint pain, history of immunosuppression, etc.)	◯	◯	◯	◯	◯

If you can think of other significant clinical indicators (i.e., clinical red flags/ signs of organic pathology) to consider in the diagnostic work up for chronic headaches, please list them here

____

What would be your course of action if a patient referred to you with chronic headache HAS significant clinical indicators (i.e., clinical red flags/ signs of organic pathology)? (*please select only one option. If none of the options listed are a part of your practice, please explain/ describe your course of action in the 'other' section)

- ◯ Deny patient with no suggestions to referring provider
- ◯ Deny patient with suggestions to referring provider
- ◯ Re-direct referral to Emergency Department
- ◯ Re-direct referral to Neurology and/ or Pediatrics and/ or other relevant specialist (as outpatient)
- ◯ Accept patient and request that referring provider complete additional workup
- ◯ Accept patient and assess yourself
- ◯ Other (will explain below)

If none of the options listed are apart of your practice, please explain/ describe your course of action here

____

Please rate the importance of completing the following laboratory investigations for patients with chronic headaches WITHOUT significant clinical indicators (i.e., clinical red flags/ clinical signs of organic pathology) prior to referral/ acceptance to your chronic pain program/ team/ clinic

	Not at all important	Somewhat important	Important	Very important	Extremely important
Serum Complete Blood Count (i.e., Hemoglobin, Hematocrit, Red Blood Cell Count, White Blood Cell Count, Platelet Count, etc.)	◯	◯	◯	◯	◯
Serum Thyroid Function (i.e., TSH, T4, etc.)	◯	◯	◯	◯	◯
Serum Electrolytes (i.e., Sodium, Potassium, Chloride, etc.)	◯	◯	◯	◯	◯

Serum Glucose	◯	◯	◯	◯	◯
Serum Calcium	◯	◯	◯	◯	◯
Serum Albumin	◯	◯	◯	◯	◯
Serum Creatinine	◯	◯	◯	◯	◯
Serum Blood Urea Nitrogen	◯	◯	◯	◯	◯
None	◯	◯	◯	◯	◯

Other (*please list any additional laboratory investigations you would consider either important, very important or extremely important to consider in the diagnostic work-up of referred patients with chronic headaches WITHOUT significant clinical indicators, i.e., clinical red flags/ clinical signs of organic pathology) ____________________

Please rate the importance of completing the following diagnostic imaging investigations for patients with chronic headache, WITHOUT significant clinical indicators (i.e., clinical red flags/ clinical signs of organic pathology) prior to referral/ acceptance to your program/ team/ clinic

	Not at all important	Somewhat important	Important	Very important	Extremely important
Computed Tomography (CT) of the head	◯	◯	◯	◯	◯
Magnetic Resonance Imaging (MRI) of the head	◯	◯	◯	◯	◯
None	◯	◯	◯	◯	◯

Other (*please list any additional diagnostic imaging investigations you would consider either important, very important or extremely important to consider in the diagnostic work-up of referred patients with chronic headaches WITHOUT significant clinical indicators, i.e., clinical red flags/ clinical signs of organic pathology) ____________________

Please rate the importance of completing the following diagnostic procedures for patients with chronic headache, WITHOUT significant clinical indicators (i.e., clinical red flags/ clinical signs of organic pathology) prior to referral/ acceptance to your program/ team/ clinic

	Not at all important	Somewhat important	Important	Very important	Extremely important
Sleep Study	◯	◯	◯	◯	◯
Lumbar Puncture	◯	◯	◯	◯	◯
Temporomandibular Joint (TMJ) Assessment	◯	◯	◯	◯	◯
Visual Acuity Examination	◯	◯	◯	◯	◯
None	◯	◯	◯	◯	◯

Other (*please list any additional diagnostic procedures you would consider either important, very important or extremely important to consider in the diagnostic work-up of referred patients with chronic headaches WITHOUT significant clinical indicators, i.e., clinical red flags/ clinical signs of organic pathology) ________________

Do you have/ know of/ use any clinical decision support tools or diagnostic algorithms for diagnosing chronic headaches (e.g., International Headache Society (HIS) international classification of headache disorders)?
- ◯ Yes
- ◯ No

If yes, please list. ________________

Please rate the importance of considering the following significant clinical indicators (i.e., clinical red flags/ clinical signs of organic pathology) in the diagnostic work-up of referred patients with Chronic Abdominal Pain.

	Not at all important	Somewhat important	Important	Very important	Extremely important
Persistent right upper quadrant pain and/ or persistent right lower quadrant pain	◯	◯	◯	◯	◯
Persistent vomiting	◯	◯	◯	◯	◯
Persistent diarrhea	◯	◯	◯	◯	◯
Bloody stools	◯	◯	◯	◯	◯
Bloody emesis	◯	◯	◯	◯	◯
Unexplained weight loss	◯	◯	◯	◯	◯
Systemic signs and symptoms (i.e., fever, rash, oral lesions, etc.)	◯	◯	◯	◯	◯
History of prior surgeries	◯	◯	◯	◯	◯
Concern or diagnosis of an eating disorder	◯	◯	◯	◯	◯

If you can think of other significant clinical indicators (i.e., clinical red flags/ signs of organic pathology) to consider in the diagnostic work up for chronic abdominal pain, please list them here. ________________

What would be your course of action if a patient referred to you HAS significant clinical indicators (i.e., clinical red flags/ clinical signs of organic pathology)? (*please select only one option. If none of the options listed are a part of your practice, please explain/ describe your course of action in the 'other' section)

- ◯ Deny patient with no suggestions to referring provider
- ◯ Deny patient with suggestions to referring provider
- ◯ Re-direct referral to Emergency Department
- ◯ Re-direct referral to Gastroenterology and/ or Pediatrics and/ or other relevant specialist (as outpatient)
- ◯ Accept patient and request referring provider complete required work-up
- ◯ Accept patient and assess yourself
- ◯ Other (will explain below)

If none of the options listed are apart of your practice, please explain/ describe your course of action here

____

Please rate the importance of completing the following laboratory investigations for patients with chronic abdominal pain, WITHOUT significant clinical indicators (i.e., clinical red flags/ clinical signs of organic pathology) prior to referral/ acceptance to your program/ team/ clinic

	Not at all important	Somewhat important	Important	Very important	Extremely important
Serum Complete Blood Count (i.e., Hemoglobin, Hematocrit, Red Blood Cell Count, White Blood Cell Count, Platelets, etc.)	◯	◯	◯	◯	◯
Serum Glucose	◯	◯	◯	◯	◯
Serum Calcium	◯	◯	◯	◯	◯
Serum Albumin	◯	◯	◯	◯	◯
Serum Creatinine	◯	◯	◯	◯	◯
Serum Blood Urea Nitrogen	◯	◯	◯	◯	◯
Serum Thyroid Function (i.e., TSH, T4, etc.)	◯	◯	◯	◯	◯
Serum Electrolytes (i.e., Sodium, Potassium, Chloride, etc.)	◯	◯	◯	◯	◯
Serum Liver Function (i.e., Alkaline Phosphatase, Alanine Amino Transferase, Aspartate Amino Transferase, Bilirubin, etc.)	◯	◯	◯	◯	◯
Serum Lipase and/ or Amylase	◯	◯	◯	◯	◯
Serum C-Reactive Protien	◯	◯	◯	◯	◯
Serum Tissue Transglutaminase (TTG)	◯	◯	◯	◯	◯
Fecal Occult Blood Test (FOBT)	◯	◯	◯	◯	◯
Fecal Culture and Sensitivity	◯	◯	◯	◯	◯
Fecal Ova and Parasite	◯	◯	◯	◯	◯

Urine Culture and Sensitivity	○	○	○	○	○
Urinalysis	○	○	○	○	○
None	○	○	○	○	○

Other (*please list any additional laboratory investigations you would consider either important, very important or extremely important to consider in the diagnostic work-up of referred patients with chronic abdominal pain WITHOUT significant clinical indicators, i.e., clinical red flags/ clinical signs of organic pathology) __________________________________

Please rate the importance of completing the following diagnostic imaging investigations for patients with chronic abdominal pain, WITHOUT significant clinical indicators (i.e., clinical red flags/ clinical signs of organic pathology) prior to referral/ acceptance to your program/ team/ clinic

	Not at all important	Somewhat important	Important	Very important	Extremely important
Abdominal X-Ray	○	○	○	○	○
Abdominal Ultrasound	○	○	○	○	○
Abdominal Computed Tomography (CT)	○	○	○	○	○
Abdominal Magnetic Resonance Imaging (MRI)	○	○	○	○	○
None	○	○	○	○	○

Other (*please list any additional diagnostic images you would consider either important, very important or extremely important to consider in the diagnostic work-up of referred patients with chronic abdominal pain WITHOUT significant clinical indicators, i.e., clinical red flags/ clinical signs of organic pathology) __________________________________

Please rate the importance of completing the following diagnostic procedures for patients with chronic abdominal pain, WITHOUT significant clinical indicators (i.e., clinical red flags/ clinical signs of organic pathology) prior acceptance to your program/ team/ clinic

	Not at all important	Somewhat important	Important	Very important	Extremely important
Hydrogen Breath Test (for fructose/ lactose sensitivities)	○	○	○	○	○
Endoscopy with biopsies	○	○	○	○	○
Endoscopy without biopsies	○	○	○	○	○

Local anesthetic injection to rule in or out Abdominal Cutaneous Nerve Entrapment Syndrome (ACNES)	○	○	○	○	○
None	○	○	○	○	○

Other (*please list any additional diagnostic procedure investigations you would consider either important, very important or extremely important to consider in the diagnostic work-up of referred patients with chronic abdominal pain WITHOUT significant clinical indicators, i.e., clinical red flags/ clinical signs of organic pathology) ____________________________________

Do you have/ know of/ use any clinical decision support tools or diagnostic algorithms for diagnosing chronic abdominal pain? (e.g., American Academy of Pediatrics Recommendations on Chronic Abdominal Pain in Children)
○ Yes
○ No

If yes, please list. ____________________________________

Please rate the importance of considering the following significant clinical indicators (i.e., clinical red flags/ clinical signs of organic pathology) in the diagnostic work-up of referred patients with Chronic Pelvic Pain.

	Not at all important	Somewhat important	Important	Very important	Extremely important
Rectal bleeding	○	○	○	○	○
Post-coital bleeding	○	○	○	○	○
Excessive or unexplained weight loss	○	○	○	○	○
Pelvic mass	○	○	○	○	○
Dysmenorrhea	○	○	○	○	○
Menorrhagia	○	○	○	○	○
Vaginal discharge	○	○	○	○	○
Tenesmus (i.e., rectal spasm)	○	○	○	○	○
Testicular mass	○	○	○	○	○
History of physical trauma	○	○	○	○	○
History of sexual trauma	○	○	○	○	○
History of congenital anomalies	○	○	○	○	○

If you can think of other significant clinical indicators (i.e., clinical red flags/ clinical signs of organic pathology) to consider in the diagnostic work up for chronic pelvic pain, please list them here. ____________________________________

What would be your course of action if a patient referred to you has significant clinical indicators (i.e., clinical red flags/ clinical signs of organic pathology). Please select only one option. If none of the options listed are a part of your practice, please explain/ describe your course of action in the 'other' section.

- ◯ Deny patient with no suggestions to referring provider
- ◯ Deny patient with suggestions to referring provider
- ◯ Re-direct referral to the Emergency Department
- ◯ Re-direct referral to Gynecology and/ or Urology and/ or Pediatrics and/ or other relevant specialist (as outpatient)
- ◯ Accept patient and request referring provider complete required work-up
- ◯ Accept patient and assess yourself
- ◯ Other (will explain below)

If none of the options listed are apart of your practice, please explain/ describe your course of action here

____

Please rate the importance of completing the following laboratory investigations for patients with Chronic Pelvic Pain, WITHOUT significant clinical indicators (i.e., clinical red flags/ clinical signs of organic pathology) prior to referral/ acceptance to your program/ team/ clinic

	Not at all important	Somewhat important	Important	Very important	Extremely important
Urinalysis	◯	◯	◯	◯	◯
Urine Culture and Sensitivity	◯	◯	◯	◯	◯
Swab for Sexually Transmitted Infections	◯	◯	◯	◯	◯
Serum or urine beta hCG	◯	◯	◯	◯	◯
Serum CBC (i.e., Hemoglobin, Hematocrit, Red Blood Cell count, White Blood Cell count, Platelets, etc.)	◯	◯	◯	◯	◯
None	◯	◯	◯	◯	◯

Other (*please list any additional laboratory investigations you would consider either important, very important or extremely important to consider in the diagnostic work-up of referred patients with chronic pelvic pain WITHOUT significant clinical indicators, i.e., clinical red flags/ clinical signs of organic pathology).

____

Please rate the importance of completing the following diagnostic imaging investigations for patients with Chronic Pelvic Pain, WITHOUT significant clinical indicators (i.e., clinical red flags/ clinical signs of organic pathology) prior to referral/ acceptance to your program/ team/ clinic

	Not at all important	Somewhat important	Important	Very important	Extremely important

Transvaginal Ultrasound (for females)	◯	◯	◯	◯	◯
Testicular Ultrasound (for males)	◯	◯	◯	◯	◯
Abdominal & Pelvis Ultrasound	◯	◯	◯	◯	◯
Computed Tomography (CT) of pelvis	◯	◯	◯	◯	◯
Magnetic Resonance Imaging (MRI) of pelvis	◯	◯	◯	◯	◯
None	◯	◯	◯	◯	◯

Other (*please list any additional diagnostic imaging investigations you would consider either important, very important or extremely important to consider in the diagnostic work-up of referred patients with chronic pelvic pain WITHOUT significant clinical indicators, i.e., clinical red flags/ clinical signs of organic pathology) __________________________________

Please rate the importance of completing the following diagnostic procedures for patients with Chronic Pelvic Pain, WITHOUT significant clinical indicators (i.e., clinical red flags/ clinical signs of organic pathology) prior to referral/ acceptance to your program/ team/ clinic

	Not at all important	Somewhat important	Important	Very important	Extremely important
Diagnostic laparoscopy	◯	◯	◯	◯	◯
Colonoscopy	◯	◯	◯	◯	◯
Barium enema	◯	◯	◯	◯	◯
Cystoscopy	◯	◯	◯	◯	◯
None	◯	◯	◯	◯	◯

Other (*please list any additional diagnostic procedure investigations you would consider either important, very important or extremely important to consider in the diagnostic work-up of referred patients with chronic pelvic pain WITHOUT significant clinical indicators, i.e., clinical red flags/ clinical signs of organic pathology) __________________________________

Do you have/ know of/ use any clinical decision support tools or diagnostic algorithms for diagnosing chronic pelvic pain? (e.g., Canadian Association of Radiologists/ Society of Obstetricians and Gynaecologists statement on performing ultrasound examinations of female pelvis)

◯ Yes
◯ No

If yes, please list. __________________________________

Please rate the importance of considering the following significant clinical indicators (i.e., clinical red flags/ clinical signs of organic pathology) in the diagnostic work-up of referred patients with chronic musculoskeletal and/ or joint pain.

	Not at all important	Somewhat important	Important	Very important	Extremely important
Arthralgia with redness and edema	○	○	○	○	○
Pain and stiffness in the morning	○	○	○	○	○
Pain at night	○	○	○	○	○
Bony tenderness	○	○	○	○	○
Unexplained weight loss	○	○	○	○	○
Systemic signs and symptoms (i.e., rash, diarrhea, etc.)	○	○	○	○	○
History of congenital anomalies	○	○	○	○	○
History of prior surgery	○	○	○	○	○
History of significant physical trauma	○	○	○	○	○
Radiculopathy	○	○	○	○	○

If you can think of other significant clinical indicators (i.e., clinical red flags/ clinical signs of organic pathology) to consider in the diagnostic work up for chronic musculoskeletal and/ or joint pain, please list them here.

____

What would be your course of action if a patient referred to you HAS significant clinical indicators (i.e, clinical red flags/ clinical signs of organic pathology). Please select only one option. If none of the options listed are a part of your practice, please explain/ describe your course of action in the 'other' section.

- ○ Deny patient with no suggestions to referring provider
- ○ Deny patient with suggestions to referring provider
- ○ Re-direct referral to Emergency Department
- ○ Re-direct referral to Rheumatology and/ or Orthopedics and/ or Pediatrics and/ or other relevant specialist (as outpatient)
- ○ Accept patient and request referring provider complete required work-up
- ○ Accept patient and assess yourself
- ○ Other (will explain below)

If none of the options listed are apart of your practice, please explain/ describe your course of action here

____

Please rate the importance of completing the following laboratory investigations for patients with chronic musculoskeletal and/ or joint pain, WITHOUT significant clinical indicators (i.e., clinical red flags/ clinical signs of organic pathology) prior to referral/ acceptance to your program/ team/ clinic

	Not at all important	Somewhat important	Important	Very important	Extremely important

Serum Complete Blood Count (i.e., Hemoglobin, Hematocrit, Red Blood Cell Count, White Blood Cell Count, Platelets, etc.)	◯	◯	◯	◯	◯
Serum Glucose	◯	◯	◯	◯	◯
Serum Calcium	◯	◯	◯	◯	◯
Serum Albumin	◯	◯	◯	◯	◯
Serum Creatinine	◯	◯	◯	◯	◯
Serum Blood Urea Nitrogen	◯	◯	◯	◯	◯
Serum Creatinine Kinase (CK)	◯	◯	◯	◯	◯
Serum Erythrocyte Sedimentation Rate (ESR)	◯	◯	◯	◯	◯
Serum C-Reactive Protein (CRP)	◯	◯	◯	◯	◯
Serum Antinuclear Antibodies (ANA)	◯	◯	◯	◯	◯
Serum Rheumatoid Factor (RF)	◯	◯	◯	◯	◯
Serum Thyroid Function (i.e., TSH, T4, etc.)	◯	◯	◯	◯	◯
Serum Tissue Transglutaminase (TTG)	◯	◯	◯	◯	◯
None	◯	◯	◯	◯	◯

Other (*please list additional laboratory investigations you would consider either extremely important, very important or important to consider in the diagnostic work-up of referred patients WITHOUT significant clinical indicators, i.e., clinical red flags/ clinical signs of organic pathology) __________________________________

Please rate the importance of completing the following diagnostic imaging investigations for patients with chronic musculoskeletal and/ or joint pain, WITHOUT significant clinical indicators (i.e., clinical red flags/ clinical signs of organic pathology) prior to referral/ acceptance to your program/ team/ clinic

	Not at all important	Somewhat important	Important	Very important	Extremely important
X-Ray of affected area(s)	◯	◯	◯	◯	◯
Ultrasound of affected area(s)	◯	◯	◯	◯	◯
Computed Tomography (CT) of affected area(s)	◯	◯	◯	◯	◯
Magnetic Resonance Imaging of affected area(s)	◯	◯	◯	◯	◯
None	◯	◯	◯	◯	◯

Other (*please list additional diagnostic imaging investigations you would consider either extremely important, very important or important to consider in the diagnostic work-up of referred patients WITHOUT significant clinical indicators, i.e., clinical red flags/ clinical signs of organic pathology) __________________________________

Please rate the importance of completing the following diagnostic procedure investigations for patients with chronic musculoskeletal and/ or joint pain, WITHOUT significant clinical indicators (i.e., clinical red flags/ clinical signs of organic pathology) prior to referral/ acceptance to your program/ team/ clinic

	Not at all important	Somewhat important	Important	Very important	Extremely important
Muscle biopsy	◯	◯	◯	◯	◯
Nerve Conduction Studies (NCS)	◯	◯	◯	◯	◯
Electromyography (EMG)	◯	◯	◯	◯	◯
None	◯	◯	◯	◯	◯

Other (*please list additional diagnostic procedure investigations you would consider either extremely important, very important or important to consider in the diagnostic work-up of referred patients WITHOUT significant clinical indicators, i.e., clinical red flags/ clinical signs of organic pathology) __________________________________

Do you have/ know of/ use any clinical decision support tools or diagnostic algorithms for diagnosing chronic musculoskeletal and/ or joint pain in children and adolescents?

◯ Yes
◯ No

If yes, please list. __________________________________

Please rate the importance of considering the following significant clinical indicators (i.e., clinical red flags/ clinical signs of organic pathology) in the diagnostic work-up of referred patients with chronic back pain.

	Not at all important	Somewhat important	Important	Very important	Extremely important
Pain unrelated to activity	◯	◯	◯	◯	◯
Unrelenting night pain	◯	◯	◯	◯	◯
Widespread neurological symptoms	◯	◯	◯	◯	◯
Bilateral sciatica	◯	◯	◯	◯	◯
Radiculopathy	◯	◯	◯	◯	◯

Incontinence (bladder and/ or bowel)	◯	◯	◯	◯	◯
Unexplained weight loss	◯	◯	◯	◯	◯
Fever or chills	◯	◯	◯	◯	◯
History of infection or trauma	◯	◯	◯	◯	◯
History of immunocompromised condition	◯	◯	◯	◯	◯
History of cancer	◯	◯	◯	◯	◯

If you can think of other significant clinical indicators (i.e., clinical red flags/ clinical signs of organic pathology) to consider in the diagnostic work up for chronic back pain, please list them here. ____________________________________

What would be your course of action if a patient referred to you HAS significant clinical indicators (i.e., clinical red flags/ clinical signs of organic pathology)? Please select only one option. If none of the options listed are a part of your practice, please explain/ describe your course of action in the 'other' section.

◯ Deny patient with no suggestions to referring provider
◯ Deny patient with suggestions to referring provider
◯ Re-direct referral to Emergency Department
◯ Re-direct referral to Orthopedics and/ or Neurology and/ or Pediatrics and/ or other relevant specialist
◯ Accept patient and request referring provider complete required work-up
◯ Accept patient and assess yourself
◯ Other (will explain below)

If none of the options listed are apart of your practice, please explain/ describe your course of action here ____________________________________

Please rate the importance of completing the following laboratory investigations for patients with chronic back pain, WITHOUT significant clinical indicators (i.e., clinical red flags/ clinical signs of organic pathology) prior to referral/ acceptance to your program/ team/ clinic

	Not at all important	Somewhat important	Important	Very important	Extremely important
Serum Complete Blood Count (i.e., Hemoglobin, Hematocrit, Red Blood Cell Count, White Blood Cell Count, Platelets, etc.)	◯	◯	◯	◯	◯
Serum Creatinine Kinase (CK)	◯	◯	◯	◯	◯
Serum C-Reactive Protein (CRP)	◯	◯	◯	◯	◯
Serum Erythrocyte Sedimentation Rate (ESR)	◯	◯	◯	◯	◯
Serum Calcium	◯	◯	◯	◯	◯
Serum Alkaline Phosphate	◯	◯	◯	◯	◯
None	◯	◯	◯	◯	◯

Other (*please list additional laboratory investigations you would consider either extremely important, very important or important to consider in the diagnostic work-up of referred patients WITHOUT significant clinical indicators, i.e., clinical red flags/ clinical signs of organic pathology) __________________________________

Please rate the importance of completing the following diagnostic imaging investigations for patients with chronic back pain, WITHOUT significant clinical indicators (i.e., clinical red flags/ clinical signs of organic pathology) prior to referral/ acceptance to your program/ team/ clinic

	Not at all important	Somewhat important	Important	Very important	Extremely important
X-Ray of affected area(s)	◯	◯	◯	◯	◯
Ultrasound of affected area(s)	◯	◯	◯	◯	◯
Computed Tomography (CT) of affected area(s)	◯	◯	◯	◯	◯
Magnetic Resonance Imaging (MRI) of affected area(s)	◯	◯	◯	◯	◯
None	◯	◯	◯	◯	◯

Other (*please list additional diagnostic imaging investigations you would consider either extremely important, very important or important to consider in the diagnostic work-up of referred patients WITHOUT significant clinical indicators, i.e., clinical red flags/ clinical signs of organic pathology) __________________________________

Would you require any diagnostic procedures to be completed before seeing the patient if the patient DOES NOT have any significant clinical indicators (i.e., clinical red flags/ clinical signs of organic pathology)?
◯ Yes
◯ No

If yes, please list __________________________________

Do you have/ know of/ use any clinical decision support tools or diagnostic algorithms for diagnosing chronic back pain in children and adolescents?
◯ Yes
◯ No

If yes, please list __________________________________

Please rate the importance of considering the following significant clinical indicators (i.e., clinical red flags/ clinical signs of organic pathology) in the diagnostic work-up of referred patients with suspected CRPS Type 1

	Not at all important	Somewhat important	Important	Very important	Extremely important
Suspicion of neuropathies	◯	◯	◯	◯	◯
Fever and/ or chills	◯	◯	◯	◯	◯
Suspicion of active bone and/ or soft tissue damage (e.g., stress fracture, ligament injury, etc.)	◯	◯	◯	◯	◯

Other (*please list additional significant clinical indicators, i.e., clinical red flags/ clinical signs of organic pathology, you would consider either extremely important, very important or important to consider in the diagnostic work-up of referred patients with suspected CRPS Type 1) __________________________________

What would be your course of action if a patient referred to you HAS significant clinical indicators (i.e., clinical red flags/ clinical signs of organic pathology)? Please select only one option. If none of the options listed are a part of your practice, please explain/ describe your course of action in the 'other' section.

- ◯ Deny patient with no suggestions to referring provider
- ◯ Deny patient with suggestions to referring provider
- ◯ Re-direct referral to Emergency Department
- ◯ Re-direct referral to Orthopedics and/ or Neurology and/ or Pediatrics and/ or other relevant specialist (as outpatient)
- ◯ Accept patient and request referring provider complete required work-up
- ◯ Accept patient and assess yourself
- ◯ Other (will explain below)

If none of the options listed are apart of your practice, please explain/ describe your course of action here __________________________________

Please rate the importance of completing the following laboratory investigations for patients with suspected CRPS Type 1, WITHOUT significant clinical indicators (i.e., clinical red flags/ clinical signs of organic pathology) prior to referral/ acceptance to your program/ team/ clinic

	Not at all important	Somewhat important	Important	Very important	Extremely important
Complete Blood Count (i.e., Hemoglobin, Hematocrit, Red Cell Count, White Cell Count, Platelets, etc.)	◯	◯	◯	◯	◯
Serum Creatinine Kinase (CK)	◯	◯	◯	◯	◯
Serum C-Reactive Protein (CRP)	◯	◯	◯	◯	◯
Serum Erythrocyte Sedimentation Rate (ESR)	◯	◯	◯	◯	◯

None	○	○	○	○	○

Other (*please list additional laboratory investigations you would consider either extremely important, very important or important to consider in the diagnostic work-up of referred patients WITHOUT significant clinical indicators, i.e., clinical red flags/ clinical signs of organic pathology) __________________________________

Please rate the importance of completing the following diagnostic imaging investigations for patients with suspected CRPS Type 1, WITHOUT significant clinical indicators (i.e., clinical red flags/ clinical signs of organic pathology) prior to referral/ acceptance to your program/ team/ clinic

	Not at all important	Somewhat important	Important	Very important	Extremely important
Duplex Ultrasonography of affected area(s)	○	○	○	○	○
Bone scan of affected area(s)	○	○	○	○	○
X-Ray of affected area(s)	○	○	○	○	○
Magnetic Resonance Imaging (MRI) of affected area(s)	○	○	○	○	○
Computed Tomography (CT) of affected area(s)	○	○	○	○	○
None	○	○	○	○	○

Other (*please list additional diagnostic imaging investigations you would consider either extremely important, very important or important to consider in the diagnostic work-up of referred patients WITHOUT significant clinical indicators, i.e., clinical red flags/ clinical signs of organic pathology) __________________________________

Please rate the importance of completing the following diagnostic procedures for patients with suspected CRPS Type 1, WITHOUT significant clinical indicators (i.e., clinical red flags/ clinical signs of organic pathology) prior to referral/ acceptance to your program/ team/ clinic

	Not at all important	Somewhat important	Important	Very important	Extremely important
Nerve Conduction Studies (NCS)	○	○	○	○	○
Local anesthetic injection trial of affected area(s)	○	○	○	○	○
None	○	○	○	○	○

Other (*please list additional diagnostic procedure investigations you would consider either extremely important, very important or important to consider in the diagnostic work-up of referred patients WITHOUT significant clinical indicators, i.e., clinical red flags/ clinical signs of organic pathology) __________________________________

Do you have/ know of/ use any clinical decision support tools or diagnostic algorithms for diagnosing CRPS in children and adolescents (e.g., Budapest criteria)?	◯ Yes<br>◯ No
If yes, please list	__________________________________
Please list what patient reported outcomes you consider important to capture/ collect in patients referred to you with any of the primary chronic pain diagnoses listed above PRIOR to acceptance into your program (e.g., Numerical Rating Scale, Brief Pain Inventory, PROMIS Pain Interference Scale, Brief Pain Inventory, BATH Adolescent Pain Questionnaire, etc.).	__________________________________
Do you have any additional comments or feedback regarding the diagnostic approach in pediatric patients with chronic pain?	__________________________________
