# Supplementary material for: Attaining expert consensus on diagnostic expectations of primary chronic pain diagnoses for patients referred to interdisciplinary pediatric chronic pain programs: A delphi study with pediatric chronic pain physicians and advanced practice nurses
Source: Front Pain Res (Lausanne). 2022 Sep 12;3:1001028. doi: 10.3389/fpain.2022.1001028 (PMC9632850; doi:10.3389/fpain.2022.1001028)
Supplement: Supplementary file 2 [file Datasheet2.pdf]

Delphi Survey Round 2: Attaining Expert Consensus on Significant Clinical Indicators and Diagnostic Approaches that are Required on Patients Prior to Acceptance into Pediatric Chronic Pain Programs

You have participated in the first round of this Delphi study as an expert in the field of pediatric chronic pain. We have since aggregated participant results and found that many survey items have met consensus. However, There are several items that we still require consensus on. We are therefore inviting you to participate in the second round of this Delphi study in attempt to attain further consenus on pending items.

As a friendly reminder, the purpose of this study is to attain expert consensus on all necessary medical diagnostic approaches and indicators that are required on patients prior to acceptance into interdisciplinary pediatric chronic pain programs for common primary chronic pain diagnoses in the pediatric population. For the purpose of this study, diagnoses will be limited to 1) Chronic headaches, 2) Chronic abdominal pain, 3) Chronic pelvic pain, 4) Chrnic musculoskeletal and/ or joint pain, 5) Chronic back pain and 6) Complex Regional Pain Syndrome (CRPS) Type 1.

---

1) Please provide your record ID which was provided in your invitation email.

____

---

2) What is your medical or nursing designation?

◯ Anesthesiologist
◯ General Practitioner
◯ Pediatrician
◯ Registered Nurse
◯ Clinical Nurse Specialist
◯ Advanced Practice Nurse
◯ Other

---

3) If your medical or nursing designation is not listed above, please provide it here

____

---

4) Do you work in an interdisciplinary pediatric chronic pain program/ clinic/ team? (*To note - we define 'interdisciplinary' as a combination of two or more clinical disciplines that work together within one team to serve the pediatric chronic pain population. Disciplines include, but are not limited to: physicians, nurses, physiotherapists, psychologists, psychiatrists, occupational therapists, pharmacists, recreational therapists, etc.)

◯ Yes
◯ No

---

5) Please state the clinical role you have within your team/ clinic/ program

____

---

6) How many years of experience do you have working with the pediatric chronic pain population?

◯ 0-5 years
◯ 5-10 years
◯ 10-20 years
◯ 20-30 years
◯ 30+ years

---

7) In what geographical location do you work?

◯ Canada
◯ United States of America
◯ Europe
◯ Australia
◯ New Zealand
◯ South America
◯ Asia
◯ Africa

8) Please indicate the province/ state within which you work ________________________________

Please rate the importance of considering the following significant clinical indicators (i.e., clinical red flags/ clinical signs of organic pathology) in the diagnostic work-up of referred patients with chronic headaches.
***Please note - Consensus has been met on 100% of items in this category from round 1 survey. Additional items listed below have been added as other important significant clinical indicators by participants during round 1 survey.**

	Not at all important	Somewhat important	Important	Very important	Extremely important
9) Vomiting	○	○	○	○	○
10) History of family neurological disease	○	○	○	○	○
11) History of cancer	○	○	○	○	○
12) History of ventriculoperitoneal (VP) shunt	○	○	○	○	○
13) History of tooth pain	○	○	○	○	○
14) Postural headache	○	○	○	○	○
15) Headache upon wakening	○	○	○	○	○
16) Weight loss/ loss of appetite	○	○	○	○	○
17) Loss of developmental milestones	○	○	○	○	○

Please rate the importance of completing the following laboratory investigations for patients with chronic headaches, WITHOUT significant clinical indicators (i.e., clinical red flags/ clinical signs of organic pathology) prior to referral/ acceptance to your program/ team/ clinic
***Please note: Consensus has been met on 87.5% of the items in this category from the round 1 survey. The items provided below represent laboratory investigations that participants have not yet met consensus on, as well as other laboratory investigations listed as important by participants in the round 1 survey.**

	Not at all important	Somewhat important	Important	Very important	Extremely important
18) Serum Thyroid Function: 1) Not at all important, n=7(31.8%). 2) Somewhat important, n=5 (22.7%). 3) Important, n=9(40.9%). 4) Very important, n=1(4.5%)	○	○	○	○	○
19) Serum Vitamin D level: Other suggestion	○	○	○	○	○
20) Serum Ferritin level: Other suggestion	○	○	○	○	○

Please rate the importance of completing the following diagnostic procedure investigations for patients with chronic headaches, WITHOUT significant clinical indicators (i.e., clinical red flags/ clinical signs of organic pathology) prior to referral/ acceptance to your program/ team/ clinic
***Please note: Consensus has been met on 75% of the items in this category from the round 1 survey. The items provided below represent diagnostic procedure investigations that participants have not yet met consensus on, as well as other laboratory investigations listed as important by participants in the round 1 survey.**

	Not at all important	Somewhat important	Important	Very important	Extremely important
21) Visual acuity examination: 1) Not at all important, n=7(31.8%). 2) Somewhat important, n=7(31.8%). 3) Important, n=4(18.2%). 4) Very important, n=3(13.6%). 5) Extremely important, n=1(4.5%)	○	○	○	○	○
22) Papilledema assessment: Other suggestion	○	○	○	○	○

Please rate the importance of considering the following significant clinical indicators (i.e., clinical red flags/ clinical signs of organic pathology) in the diagnostic work-up of referred patients with chronic abdominal pain.
***Please note - Consensus has been met on 100% of items in this category from round 1 survey. Additional items listed below have been added as other important significant clinical indicators by participants during round 1 survey.**

	Not at all important	Somewhat important	Important	Very important	Extremely important
23) Pain that wakes from sleep: Other suggestion	○	○	○	○	○
24) Family history of a GI cancer: Other suggestion	○	○	○	○	○
25) History of trauma: Other suggestion	○	○	○	○	○
26) Referred back pain: Other suggestion	○	○	○	○	○
27) Bilious emesis: Other suggestion	○	○	○	○	○

Please rate the importance of completing the following laboratory investigations for patients with chronic abdominal pain, WITHOUT significant clinical indicators (i.e., clinical red flags/ clinical signs of organic pathology) prior to referral/ acceptance to your program/ team/ clinic *Please note: Consensus has been met on 13% of the items in this category from the round 1 survey. The items provided below represent laboratory investigations that participants have not yet met consensus on, as well as additional other laboratory investigations listed as important by participants in the round 1 survey.

		Not at all important	Somewhat important	Important	Very important	Extremely important
28)	Serum Complete Blood Cell Count: 1) Not at all important, n=3(13.6%). 2) Somewhat important, n=10(45.5%). 3) Important, n=4(18.2%). 4) Very important, n=2 (9.1%). 5) Extremely important, n=3(13.6%)	○	○	○	○	○
29)	Serum Glucose: 1) Not at all important, n=8(36.4%). 2) Somewhat important, n=7(31.8%). 3) Important, n=6(27.3%). 4) Very Important, n=0. 5) Extremely Important, n=1(4.5%)	○	○	○	○	○
30)	Serum Albumin: 1) Not at all important, n=6(27.3%). 2) Somewhat important, n=6(27.3%). 3) Important, n=7(31.8%). 4) Very important, n=2(9.1%). 5) Extremely important, n=1(4.5%)	○	○	○	○	○
31)	Serum Creatinine: 1) Not at all important, n=6(27.3%). 2) Somewhat important, n=7(31.8%). 3) Important, n=7(31.8%). 4) Very important, n=1(4.5%). 5) Extremely important, n=1(4.5%)	○	○	○	○	○
32)	Serum Blood Urea Nitrogen: 1) Not at all important, n=6(27.3%). 2) Somewhat important, n=7(31.8%). 3) Important, n=7(31.8%). 4) Very important, n=1(4.5%). 5) Extremely important, n=1(4.5%)	○	○	○	○	○

33)

Serum Thyroid Function: 1) Not at all important, n=7(31.8). 2) Somewhat important, n=7(31.8). 3) Important, n=7(31.8). 4) Very important, n=0. 5) Extremely important, n=1(4.5%) ○ ○ ○ ○ ○

34) Serum Electrolytes: 1) Not at all important, n=8(36.4). 2) Somewhat important, n=5(22.7). 3) Important, n=7(31.8). 4) Very important, n=2(9.1). 5) Extremely important, n=0 ○ ○ ○ ○ ○

35) Serum Liver Function: 1) Not at all important, n=2(9.1%). 2) Somewhat important, n=8(36.4%). 3) Important, n=6(27.3%). 4) Very important, n=4(18.2%). 5) Extremely important, n=2(9.1%) ○ ○ ○ ○ ○

36) Serum Lipase/Amylase: 1) Not at all important, n=3(13.6%). 2) Somewhat important, n=7(31.8%). 3) Important, n=6(27.3%). 4) Very important, n=6(27.3%). 5) Extremely important, n=0 ○ ○ ○ ○ ○

37) Serum C-Reactive Protein: 1) Not at all important, n=5(22.7%). 2) Somewhat important, n=8(36.4%). 3) Important, n=5(22.7%). 4) Very important, n=3(13.6%). 5) Extremely important, n=1(4.5%) ○ ○ ○ ○ ○

38) Serum Tissue Transglutaminase: 1) Not at all important, n=6(27.3%). 2) Somewhat important, n=5(22.7%). 3) Important, n=7(31.8%). 4) Very important, n=2(9.1%). 5) Extremely important, n=2(9.1%) ○ ○ ○ ○ ○

39)

		Not at all important	Somewhat important	Important	Very important	Extremely important
	Fecal Occult Blood Test: 1) Not at all important, n=7(31.8%). 2) Somewhat important, n=5(22.7%). 3) Important, n=5(22.7%). 4) Very important, n=4(18.2%). 5) Extremely important, n=1(4.5%)	○	○	○	○	○
40)	Fecal Culture & Sensitivity: 1) Not at all important, n=9(40.9%). 2) Somewhat important, n=6(27.3%). 3) Important, n=4(18.2%). 4) Very important, n=2(9.1%). 5) Extremely important, n=1(4.5%)	○	○	○	○	○
41)	Fecal Ova & Parasite:1) Not at all important, n=9(40.9%). 2) Somewhat important, n=6(27.3%). 3) Important, n=4(18.2%). 4) Very important, n=2(9.1%). 5) Extremely important, n=1(4.5%	○	○	○	○	○
42)	Urinalysis: 1) Not at all important, n=7(31.8%). 2) Somewhat important, n=8(36.4%). 3) Important, n=5(22.7%). 4) Very important, n=1(4.5%). 5) Extremely important, n=1(4.5%)	○	○	○	○	○
43)	H. Pylori screen: Other suggestion	○	○	○	○	○
44)	Fecal calprotectin: Other suggestion	○	○	○	○	○
45)	Serum Erythrocyte Sedimentation Rate: Other suggestion	○	○	○	○	○

Please rate the importance of completing the following diagnostic imaging investigations for patients with chronic abdominal pain, WITHOUT significant clinical indicators (i.e., clinical red flags/ clinical signs of organic pathology) prior to referral/ acceptance to your program/ team/ clinic

***Please note: Consensus has been met on 50% of the items in this category from the round 1 survey. The items provided below represent diagnostic imaging investigations that participants have not yet met consensus on. No other important diagnostic investigations were listed as important by participants in this category from round 1 survey.**

	Not at all important	Somewhat important	Important	Very important	Extremely important
46)					

	Not at all important	Somewhat important	Important	Very important	Extremely important
Abdominal X-Ray: 1) Not at all important, n=11(50%). 2) Somewhat important, n=4(18.2%). 3) Important, n=3(13.6%). 4) Very important, n=3(13.6%). 5) Extremely important, n=1(4.5%)	○	○	○	○	○
47) Abdominal Ultrasound: 1) Not at all important, n=1(4.5%). 2) Somewhat important, n=14(63.6%). 3) Important, n=4(18.2%). 4) Very important, n=3(13.6%). 5) Extremely important, n=0	○	○	○	○	○

Please rate the importance of completing the following diagnostic procedure investigations for patients with chronic abdominal pain, WITHOUT significant clinical indicators (i.e., clinical red flags/ clinical signs of organic pathology) prior to referral/ acceptance to your program/ team/ clinic
***Please note: Consensus has been met on 100% of the items in this category from the round 1 survey. The items provided below represent other diagnostic imaging investigations that participants listed as important during round 1 survey**

	Not at all important	Somewhat important	Important	Very important	Extremely important
48) Gastric emptying study: Other suggestion	○	○	○	○	○

Please rate the importance of considering the following significant clinical indicators (i.e., clinical red flags/ clinical signs of organic pathology) in the diagnostic work-up of referred patients with chronic pelvic pain.
***Please note - Consensus has been met on 100% of items in this category from round 1 survey. Additional items listed below have been added as other important significant clinical indicators by participants during round 1 survey.**

	Not at all important	Somewhat important	Important	Very important	Extremely important
49) Dyspareunia (pain with intercourse): Other suggestion	○	○	○	○	○
50) Hematuria: Other suggestion	○	○	○	○	○

Please rate the importance of completing the following laboratory investigations for patients with chronic pelvic pain, WITHOUT significant clinical indicators (i.e., clinical red flags/ clinical signs of organic pathology) prior to referral/ acceptance to your program/ team/ clinic *Please note: Consensus has been met on 0% of the items in this category from the round 1 survey. The items provided below represent laboratory investigations that participants have not yet met consensus on. No additional important laboratory investigations were listed by participants in this category during round 1 survey

		Not at all important	Somewhat important	Important	Very important	Extremely important
51)	Urinalysis: 1) Not at all important, n=5(22.7%). 2) Somewhat important, n=4(18.2%). 3) Important, n=6(27.3%). 4) Very important, n=1(4.5%). 5) Extremely important, n=6(27.3%)	○	○	○	○	○
52)	Urine Culture & Sensitivity: 1) Not at all important, n=4(18.2%). 2) Somewhat important, n=6(27.3%). 3) Important, n=6(27.3%). 4) Very important, n=2(9.1%). 5) Extremely important, n=4(18.2%)	○	○	○	○	○
53)	Swab for Sexually Transmitted Infections: 1) Not at all important, n=3(13.6%). 2) Somewhat important, n=8(36.4%). 3) Important, n=3(13.6%). 4) Very important, n=2(9.1%). 5) Extremely important, n=6(27.3%)	○	○	○	○	○
54)	Serum or Urine beta hCG: 1) Not at all important, n=4(18.2%). 2) Somewhat important, n=5(22.7%). 3) Important, n=4(18.2%). 4) Very important, n=3(13.6%). 5) Extremely important, n=6(27.3%)	○	○	○	○	○
55)	Serum Complete Blood Cell count: 1) Not at all important, n=4(18.2%). 2) Somewhat important, n=6(27.3%). 3) Important, n=8(36.4%). 4) Very important, n=0. 5. Extremely important, n=4(18.2%)	○	○	○	○	○

Please rate the importance of completing the following diagnostic imaging investigations for patients with chronic pelvic pain, WITHOUT significant clinical indicators (i.e., clinical red flags/ clinical signs of organic pathology) prior to referral/ acceptance to your program/ team/ clinic
***Please note: Consensus has been met on 40% of the items in this category from the round 1 survey. The items provided below represent diagnostic imaging investigations that participants have not yet met consensus on. No additional diagnostic imaging investigations were listed by participants in this category during round 1 survey**

	Not at all important	Somewhat important	Important	Very important	Extremely important
56) Testicular ultrasound: 1) Not at all important, n=9(40.9%). 2) Somewhat important, n=5(22.7%). 3) Important, n=4(18.2%). 4) Very important, n=2(9.1%). 5) Extremely important, n=2(9.1%)	○	○	○	○	○
57) Abdominal & Pelvis ultrasound: 1) Not at all important, n=8(36.4%). 2) Somewhat important, n=6(27.3%). 3) Important, n=3(13.6%). 4) Very important, n=3(13.6%). 5) Extremely important, n=2(9.1%)	○	○	○	○	○

Please rate the importance of considering the following significant clinical indicators (i.e., clinical red flags/ clinical signs of organic pathology) in the diagnostic work-up of referred patients with chronic musculoskeletal and/or joint pain.
***Please note - Consensus has been met on 90% of items in this category from round 1 survey. The items provided below represent significant clinical indicators that participants have not yet met consensus on, as well as other laboratory investigations listed as important by participants in the round 1 survey.**

	Not at all important	Somewhat important	Important	Very important	Extremely important
58) History of prior surgeries: 1) Not at all important, n=2(9.1%). 2) Somewhat important, n=6(27.3%). 3) Important, n=5(22.7%). 4) Very important, n=7(31.8%). 5) Extremely important, n=2(9.1%)	○	○	○	○	○
59) History of cancer: Other suggestion	○	○	○	○	○

60)

		Not at all important	Somewhat important	Important	Very important	Extremely important
	Positive trigger points: Other suggestion	○	○	○	○	○
61)	Known Ehlers-Danlos Syndrome (EDS): Other suggestion	○	○	○	○	○

Please rate the importance of completing the following laboratory investigations for patients with chronic musculoskeletal and/ or joint pain, WITHOUT significant clinical indicators (i.e., clinical red flags/ clinical signs of organic pathology) prior to referral/ acceptance to your program/ team/ clinic
***Please note: Consensus has been met on 38% of the items in this category from the round 1 survey. The items provided below represent laboratory investigations that participants have not yet met consensus on, as well as additional items listed by participants that were considered important during the round 1 survey.**

		Not at all important	Somewhat important	Important	Very important	Extremely important
62)	Serum Complete Blood Cell Count: 1) Not at all important, n=5(22.7%). 2) Somewhat important, n=8(36.4%). 3) Important, n=4(18.2%). 4) Very important, n=3(13.6%). 5) Extremely important, n=2(9.1%)	○	○	○	○	○
63)	Serum Blood Urea Nitrogen: 1) Not at all important, n=11(50%). 2) Somewhat important, n=4(18.2%). 3) Important, n=6(27.3%). 4) Very important, n=1(4.5%). 5) Extremely important, n=0	○	○	○	○	○
64)	Serum Creatinine Kinase: 1) Not at all important, n=6 (27.3%). 2) Somewhat important, n=4(18.2%). 3) Important, n=7(31.8%). 4) Very important, n=3(13.6%). 5) Extremely important, n=2(9.1%)	○	○	○	○	○
65)	Serum C-Reactive Protein: 1) Not at all important, n=6 (27.3%). 2) Somewhat important, n=5(22.7%). 3) Important, n=5(22.7%). 4) Very important, n=4(18.2%). 5) Extremely important, n=2(9.1%)	○	○	○	○	○

66)

	Not at all important	Somewhat important	Important	Very important	Extremely important
Serum Antinuclear Antibodies: 1) Not at all important, n=5 (22.7%). 2) Somewhat important, n=9 (40.9%). 3) Important, n=2 (9.1%). 4) Very important, n=5 (22.7%). 5) Extremely important, n=1 (4.5%)	○	○	○	○	○
67) Serum Rheumatoid Factor:1) Not at all important, n=5 (22.7%). 2) Somewhat important, n=9 (40.9%). 3) Important, n=2 (9.1%). 4) Very important, n=5 (22.7%). 5) Extremely important, n=1 (4.5%)	○	○	○	○	○
68) Serum Thyroid Function: 1) Not at all important, n=7 (31.8%). 2) Somewhat important, n=6 (27.3%). 3) Important, n=6 (27.3%). 4) Very important, n=2 (9.1%). 5) Extremely important, n=1 (4.5%)	○	○	○	○	○
69) Serum HLA B27: Other suggestion	○	○	○	○	○
70) Serum Vitamin D: Other suggestion	○	○	○	○	○
71) Serum Vitamin B12: Other suggestion	○	○	○	○	○
72) Serum Folate: Other suggestion	○	○	○	○	○
73) Serum Complement Levels: Other suggestion	○	○	○	○	○
74) Urinalysis: Other suggestion	○	○	○	○	○

Please rate the importance of completing the following diagnostic imaging investigations for patients with chronic musculoskeletal and/ or joint pain, WITHOUT significant clinical indicators (i.e., clinical red flags/ clinical signs of organic pathology) prior to referral/ acceptance to your program/ team/ clinic
***Please note: Consensus has been met on 25% of the items in this category from the round 1 survey. The items provided below represent diagnostic imaging investigations that participants have not yet met consensus on. No additional diagnostic imaging investigations were listed by participants in this category during round 1 survey**

	Not at all important	Somewhat important	Important	Very important	Extremely important
75)					

	Not at all important	Somewhat important	Important	Very important	Extremely important
X-Ray of affected area(s): 1) Not at all important, n=6(27.3%). 2) Somewhat important, n=3 (13.6%). 3) Important, n=5(22.7%). 4) Very important, n=5(22.7%). 5) Extremely important, n=3(13.6%)	○	○	○	○	○
76) Ultrasound of affected area(s): 1) Not at all important, n=7(31.8%). 2) Somewhat important, n=7(31.8%). 3) Important, n=6(27.3%). 4) Very important, n=2(9.1%). 5) Extremely important, n=0	○	○	○	○	○
77) Magnetic Resonance Imaging of affected area(s): 1) Not important, n=7(31.8%). 2) Somewhat important, n=6(27.3%). 3) Important, n=6(27.3%). 4) Very important, n=3(13.6%). 5) Extremely important, n=0.	○	○	○	○	○

Please rate the importance of considering the following significant clinical indicators (i.e., clinical red flags/ clinical signs of organic pathology) in the diagnostic work-up of referred patients with chronic back pain.
***Please note - Consensus has been met on 90% of items in this category from round 1 survey. The items provided below represent significant clinical indicators that participants have not yet met consensus on, as well as other items listed as important by participants in the round 1 survey.**

	Not at all important	Somewhat important	Important	Very important	Extremely important
78) Pain unrelated to activity: 1) Not at all important, n=2(9.1%). 2) Somewhat important, n=6(27.3%). 3) Important, n=8(36.4%). 4) Very important, n=4(18.2%). 5) Extremely important, n=2(9.1%)	○	○	○	○	○
79) Constant pain: Other suggestion	○	○	○	○	○
80) Redness and edema at painful site: Other suggestion	○	○	○	○	○
81) History of scoliosis: Other suggestion	○	○	○	○	○

Please rate the importance of completing the following laboratory investigations for patients with chronic back pain, WITHOUT significant clinical indicators (i.e., clinical red flags/ clinical signs of organic pathology) prior to referral/ acceptance to your program/ team/ clinic *Please note: Consensus has been met on 50% of the items in this category from the round 1 survey. The items provided below represent laboratory investigations that participants have not yet met consensus on, as well as additional items listed by participants that were considered important during the round 1 survey.

		Not at all important	Somewhat important	Important	Very important	Extremely important
82)	Serum Complete Blood Cell Count: 1) Not at all important, n=9(40.9%). 2) Somewhat important, n=6(27.3%). 3) Important, n=6(27.3%). 4) Very important, n=1(4.5%). 5) Extremely important, n=0	○	○	○	○	○
83)	Serum C-Reactive Protein: 1) Not at all important, n=6(27.3%). 2) Somewhat important, n=8(36.4%). 3) Important, n=5(22.7%). 4) Very important, n=2(9.1%). 5) Extremely important, n=0	○	○	○	○	○
84)	Serum Erythrocyte Sedimentation Rate: 1) Not at all important, n=6(27.3%). 2) Somewhat important, n=9(40.9%). 3) Important, n=5(22.7%). 4) Very important, n=2(9.1%). 5) Extremely important, n=0	○	○	○	○	○
85)	Serum Antinuclear Antibody: Other suggestion	○	○	○	○	○

Please rate the importance of completing the following diagnostic imaging investigations for patients with chronic back pain, WITHOUT significant clinical indicators (i.e., clinical red flags/ clinical signs of organic pathology) prior to referral/ acceptance to your program/ team/ clinic *Please note: Consensus has been met on 50% of the items in this category from the round 1 survey. The items provided below represent diagnostic imaging investigations that participants have not yet met consensus on. No additional diagnostic imaging investigations were listed by participants in this category during round 1 survey

		Not at all important	Somewhat important	Important	Very important	Extremely important
86)						

	Not at all important	Somewhat important	Important	Very important	Extremely important
X-Ray of affected area(s): 1) Not at all important, n=5(22.7%). 2) Somewhat important, n=5(22.7%). 3) Important, n=7(31.8%). 4) Very important, n=2(9.1%). 5) Extremely important, n=3(13.6%)	○	○	○	○	○
87) Magnetic Resonance Imaging of affected area(s): 1) Not at all important, n=6(27.3%). 2) Somewhat important, n=5(22.7%). 3) Important, n=7(31.8%). 4) Very important, n=3(13.6%). 5) Extremely important, n=1(4.5%)	○	○	○	○	○

Please rate the importance of considering the following significant clinical indicators (i.e., clinical red flags/ clinical signs of organic pathology) in the diagnostic work-up of referred patients with Complex Regional Pain Syndrome (CRPS), Type 1.
***Please note - Consensus has been met on 100% of items in this category from round 1 survey. Additional items listed below have been added as other important significant clinical indicators by participants during round 1 survey.**

	Not at all important	Somewhat important	Very important	Extremely important
88) Neurovascular changes: Other suggestion	○	○	○	○
89) History of surgery: Other suggestion	○	○	○	○
90) History of trauma: Other suggestion	○	○	○	○
91) Concern of pulselessness: Other suggestion	○	○	○	○

Please rate the importance of completing the following diagnostic imaging investigations for patients with Complex Regional Pain Syndrome (CRPS), Type 1, WITHOUT significant clinical indicators (i.e., clinical red flags/ clinical signs of organic pathology) prior to referral/ acceptance to your program/ team/ clinic
***Please note: Consensus has been met on 60% of the items in this category from the round 1 survey. The items provided below represent diagnostic imaging investigations that participants have not yet met consensus on. No additional diagnostic imaging investigations were listed by participants in this category during round 1 survey**

	Not at all important	Somewhat important	Important	Very important	Extremely important
92)					

X-Ray of affected area(s): 1) Not at all important, n=5(22.7%). 2) Somewhat important, n=7(31.8%). 3) Important, n=4(18.2%). 4) Very important, n=2(9.1%). 5) Extremely important, n=2(9.1%) ○ ○ ○ ○ ○

93) Magnetic Resonance Imaging of affected area(s): 1) Not at all important, n=9(40.9%). 2) Somewhat important, n=5(22.7%). 3) Important, n=4(18.2%). 4) Very important, n=2(9.1%). 5) Extremely important, n=2(9.1%) ○ ○ ○ ○ ○

---

94) Do you have any additional comments or feedback regarding the results from the round 1 Delphi survey? ____________________________
